# Comparative Transcriptome and MicroRNA Profiles of Equine Mesenchymal Stem Cells, Fibroblasts, and Their Extracellular Vesicles

**DOI:** 10.3390/genes16080936

**Published:** 2025-08-05

**Authors:** Sebastian Sawicki, Monika Bugno-Poniewierska, Jakub Żurowski, Tomasz Szmatoła, Ewelina Semik-Gurgul, Michał Bochenek, Elżbieta Karnas, Artur Gurgul

**Affiliations:** 1 Department of Animal Reproduction, Anatomy and Genomics, University of Agriculture in Krakow, Mickiewicza 24/28, 30-059 Krakow, Poland; monika.bugno-poniewierska@urk.edu.pl; 2 Department of Basic Sciences, University of Agriculture in Krakow, Redzina 1C, 30-248 Krakow, Poland; kubazurowski@gmail.com (J.Ż.); tomasz.szmatola@urk.edu.pl (T.S.); 3 Department of Animal Molecular Biology, National Research Institute of Animal Production, Krakowska 1 St., 32-083 Balice, Poland; ewelina.semik@iz.edu.pl; 4 Institute of Zoology and Biomedical Research, Jagiellonian University in Krakow, Gronostajowa 9, 30-387 Krakow, Poland; michal.bochenek@uj.edu.pl; 5 Department of Cell Biology, Faculty of Biochemistry, Biophysics and Biotechnology, Jagiellonian University, Gronostajowa 7, 30-387 Krakow, Poland; e.karnas@uj.edu.pl

**Keywords:** MSC, extracellular vesicles, transcriptome, miRNA, RNAseq

## Abstract

**Background:** Mesenchymal stem cells (MSCs) are a promising tool in regenerative medicine due to their ability to secrete paracrine factors that modulate tissue repair. Extracellular vesicles (EVs) released by MSCs contain bioactive molecules (e.g., mRNAs, miRNAs, proteins) and play a key role in intercellular communication. **Methods:** This study compared the transcriptomic profiles (mRNA and miRNA) of equine MSCs derived from adipose tissue (AT-MSCs), bone marrow (BM-MSCs), and ovarian fibroblasts (as a differentiated control). Additionally, miRNAs present in EVs secreted by these cells were characterized using next-generation sequencing. **Results:** All cell types met ISCT criteria for MSCs, including CD90 expression, lack of MHC II, trilineage differentiation, and adherence. EVs were isolated using ultracentrifugation and validated with nanoparticle tracking analysis and flow cytometry (CD63, CD81). Differential expression analysis revealed distinct mRNA and miRNA profiles across cell types and their secreted EVs, correlating with tissue origin. BM-MSCs showed unique regulation of genes linked to early development and osteogenesis. EVs contained diverse RNA species, including miRNA, mRNA, lncRNA, rRNA, and others. In total, 227 and 256 mature miRNAs were detected in BM-MSCs and AT-MSCs, respectively, including two novel miRNAs per MSC type. Fibroblasts expressed 209 mature miRNAs, including one novel miRNA also found in MSCs. Compared to fibroblasts, 60 and 92 differentially expressed miRNAs were identified in AT-MSCs and BM-MSCs, respectively. **Conclusions:** The results indicate that MSC tissue origin influences both transcriptomic profiles and EV miRNA content, which may help to interpret their therapeutic potential. Identifying key mRNAs and miRNAs could aid in future optimizing of MSC-based therapies in horses.

## 1. Introduction

Mesenchymal stem cells (MSCs) are multipotent progenitor cells considered promising tools utilized in regenerative medicine for humans and animals [1,2]. MSCs can be obtained from fetal tissues (umbilical cord, umbilical cord blood, and placenta) as well as from various adult tissues, of which bone marrow and adipose tissue hold the greatest practical significance due to their relative accessibility and the high content of these cells. MSCs participate in inflammatory, proliferative, remodeling, and tissue repair processes [3]. Considering the significant potential and involvement of MSCs in immunomodulation, they possess remarkable potential in veterinary medicine, especially in horses [4]. The ability of MSCs to participate in regenerative processes can be associated with their transplantation into damaged tissue and/or the production of molecules and mediators capable of activating internal repair processes [5].

Cell cultures derived from various tissue sources typically exhibit different degrees of cellular heterogeneity. The heterogeneity of MSCs is traditionally assessed based on morphology, surface marker expression, cell kinetics, differentiation potential, as well as selected gene expression patterns [6]. However, the transcriptional heterogeneity of MSCs, especially those obtained from different tissues, remains largely unexplored, and its contribution to the therapeutic value of MSCs is poorly understood.

MSCs secrete numerous paracrine factors that support the differentiation of endogenous progenitor cells, promote angiogenesis, and modulate immune responses [7]. These factors primarily include bioactive molecules such as proteins, cytokines, growth factors, as well as nucleic acids [8]. This complex array of secreted factors is mainly involved in intercellular communication [9].

Part of the paracrine secretome of MSCs comprises extracellular vesicles (EVs) [10]. EVs are a heterogeneous group of cell-derived membranous structures comprising exosomes and microvesicles that originate from the endosomal system or are shed from the plasma membrane, respectively [11]. They are biologically active molecules containing genetic material, proteins, and lipids originating from the stem cell [12,13]. According to the latest findings of MISEV2018 (Minimal Information for Studies of Extracellular Vesicles 2018), extracellular vesicles can be classified based on their size (“small EVs” < 100 nm or <200 nm, “medium/large EVs” > 200 nm), biochemical composition, or origin [14]. Due to several biogenesis pathways, extracellular vesicles can be divided into several types. Exosomes are formed by inward budding of endosomal membranes, whereas microvesicles are formed by outward budding of the plasma membrane. Apoptotic bodies are formed during apoptosis [15].

Compared to cells, extracellular vesicles have lower immunogenicity and can cross biological barriers [4]. EVs perform various functions, including participating in regenerative processes, enhancing proliferation, and also participating in cell apoptosis [16,17]. They also engage in intercellular communication through interaction and fusion with the lipid membranes of target cells, enabling the delivery of proteins and genetic information into the target cells [18].

A significant portion of EV content comprises nucleic acids, mainly in the form of RNA. Types of RNA particles present in EVs primarily include tRNA, mRNA, miRNA, and long non-coding RNA, with miRNA being the major fraction [19]. The membrane of extracellular vesicles effectively protects the cargo content (miRNA, mRNA) from RNase present in body fluids, thus miRNA derived from EVs is more stable than extracellular circulating miRNA [20].

In recent years, studies focusing on comparative transcriptome analysis of MSCs derived from different sources (bone marrow, adipose tissue, etc.) have emerged in humans [21,22], dogs [23], pigs [24], as well as studies focusing on cells undergoing differentiation [25,26]. Research has shown that each type of MSCs exhibited a unique expression pattern, which may indicate a direction for therapeutic application and enable targeted treatment.

The horse is not only an ideal large animal for obtaining donor-matched MSCs from various tissues for in vitro transcriptomic and functional studies, but it is also a species of significant physiological relevance that can be utilized for in vivo studies assessing the activity of MSCs.

In the scientific literature, there is no information regarding differences in transcriptomic profiles between MSCs derived from adipose tissue and bone marrow, as well as fibroblasts in horses. So far, many types of EVs from various tissues have been characterized [11]; however, little is known about the differences between equine EVs derived from different types of MSCs and the significance of these differences for the paracrine properties of EVs.

To the best of our knowledge, the published literature lacks data regarding the comparative transcriptomic characterization of MSCs derived from adipose tissue and bone marrow, as well as differences in miRNA cargo in extracellular vesicles isolated from these cells. As an additional cell type, ovarian fibroblasts were included to demonstrate the differences between both types of MSCs and fully differentiated cells with a similar phenotype. Mesenchymal stromal cells are utilized based on their promising features. Similarly, fibroblasts can also be considered as a therapeutic tool because of their similarities with MSCs, such as immunomodulatory properties [27]. Fibroblasts originate from the mesenchyme and exhibit high inter- and intra-organ heterogeneity, primarily reflecting differences in the expression of extracellular matrix components [28]. However, their heterogeneity has not been thoroughly analyzed.

A research hypothesis postulated that despite common significant biological properties, the transcriptome of mesenchymal stem cells varies depending on their source. It was also assumed that mesenchymal stem cells from different sources would secrete populations of extracellular vesicles with distinct miRNA cargo specific to each cell type.

The aim of the study was to compare the transcriptome of equine mesenchymal stem cells and fibroblasts, as well as the miRNA cargo contained in extracellular vesicles secreted by these cells derived from adipose tissue and bone marrow using next-generation sequencing technique.

## 2. Materials and Methods

The collection of adipose tissue, isolation, and identification of AT-MSCs and EV-MSCs (differentiation, flow cytometry, NTA) were performed using a previously validated and described method [29].

### 2.1. Experiment Design

The study utilized adipose tissue, bone marrow, and ovaries obtained from three unregistered mares of unknown age and breed. Samples were collected post-mortem at a commercial horse slaughterhouse in accordance with EU regulations. Therefore, approval from the local ethics committee for sample collection was not required. MSC lines were derived from adipose tissue and bone marrow, while a fibroblast line was derived from ovarian fragments. EVs were obtained from the cell culture supernatant for each culture using ultracentrifugation. Cells from each replicate (sample) were used to isolate total RNA. Total RNA, predominantly consisting of miRNA, was isolated from EVs. The obtained RNA was then used to prepare libraries and subjected to next-generation sequencing on the Illumina platform. Finally, a comparative analysis of the transcriptomes of individual cell types and the EVs secreted by them was conducted.

### 2.2. Isolation and Identification of BM-MSCs and AT-MSCs

The collection of adipose tissue, isolation, and identification of AT-MSCs and EV-MSCs (differentiation, flow cytometry, NTA) were validated and described in our previous work [29].

The bone marrow was posthumously extracted from the tibial bone of three individuals using a sterile needle into sterile tubes with 1000 U/mL heparin. The secured material was transported to the laboratory within 4 h of collection at 4 °C. To the bone marrow, 2 mL of previously prepared medium (DMEM 1 g/L glucose, L-glutamine, sodium bicarbonate, and phenol red) (Sigma-Aldrich, Darmstadt, Germany) supplemented with 1% penicillin/streptomycin solution (Sigma-Aldrich, Darmstadt, Germany) and 0.1% amphotericin (Sigma-Aldrich, Darmstadt, Germany) was added.

AT-MSCs were isolated from adipose tissue using collagenase type I at 37 °C for 3 h [29].

In brief, the released cell suspensions from both bone marrow and adipose tissue were filtered through a cell strainer (70 µm) (Corning) and then centrifuged at 400× *g* for 10 min. The resulting cell pellet was washed several times with sterile PBS and then cells were incubated in sterile DMEM medium supplemented with 10% FBS and 1% penicillin/streptomycin at 37 °C in a humidified atmosphere containing 5% CO_2_. The culture medium was changed every three days until cell confluence of ≥80% was achieved. The cells were passaged and frozen at −80 °C for storage.

MSCs derived from adipose tissue and bone marrow were cultured to induce adipogenic, osteogenic, and chondrogenic differentiation in a monolayer. The differentiation was confirmed using Oil Red O staining for adipogenesis (Sigma-Aldrich), Safranin O (0.1%) for chondrogenesis (Sigma-Aldrich), and Alizarin Red (2%, pH 4.2) for osteogenesis (Sigma-Aldrich).

Cells (P4) were cultured to about 80% confluence and then collected for flow cytometry. Surface antigens staining was performed for 30 min with primary antibodies against CD90 (5E10; Thermo Fisher Scientific, Waltham, MA, USA #A15726) and MHC II (CVS20; Thermo Fisher Scientific #MA5-28491), both of which were commercially conjugated. After staining and two PBS washes, the cells were resuspended in Dulbecco’s phosphate-buffered saline (dPBS) (Sigma-Aldrich, Darmstadt, Germany) and kept at 4 °C until further analysis. As a minimum 10,000 cells from each sample were analysed using a Navios flow cytometer (Beckman Coulter, Brea, CA, USA). The data were analysed using Kaluza 2.1 software.

### 2.3. Ovaries Harvesting and Fibroblast Isolation

The ovaries were posthumously obtained from three individuals. The secured material was transported to the laboratory within 4 h of collection at 4 °C. The fibroblast cell line was obtained by culturing explants by altering media [30]. The mare’s ovaries were thoroughly rinsed multiple times with sterile PBS, then cut into small fragments. These fragments were placed in culture dishes and covered with sterile culture medium (DMEM with 1000 mg/L glucose, L-glutamine, sodium bicarbonate, and phenol red) from Sigma-Aldrich, Darmstadt, Germany. The medium was further supplemented with 1% penicillin/streptomycin solution and 0.1% amphotericin (Sigma-Aldrich, Darmstadt, Germany). The mixture was distributed into four culture dishes and incubated at 37 °C in a humidified atmosphere containing 5% CO_2_. The culture medium was changed every three days until a cell confluence of ≥80% was achieved. The cells were passaged and frozen at −80 °C for storage.

### 2.4. EV Isolation and Identification

Equine AT-MSCs, BM-MSCs, and fibroblasts (P4) were expanded in culture medium as previously described [29]. Briefly, cells were washed with sterile dPBS, and the medium was replaced with exosome-depleted medium. The culture was maintained for 72 h, after which the supernatant was collected and subjected to initial centrifugation steps at 4 °C (2000× *g* for 10 min, followed by 10,000× *g* for 30 min). The supernatant was then filtered through a 0.2 μm filter (Corning). Subsequently, the supernatant underwent two rounds of ultracentrifugation using a fixed-angle rotor (70 Ti-Beckman) at 120,000× *g* for 70 min at 4 °C. After ultracentrifugation, EVs were resuspended in dPBS (Sigma-Aldrich, Darmstadt, Germany) and frozen at −80 °C for later use.

To identify the obtained EVs, nanoparticle tracking analysis (NTA) was performed using NanoSight NS500 (Malvern Instruments Ltd., Malvern, UK). The concentrations and size of EVs in the culture medium after ultracentrifugation were determined. The data were analyzed using NTA 3.2 Dev Build 3.2.16 (Malvern) analytical software and presented as mean ± standard deviation from three experimental replicates.

Using the data obtained from the NTA analysis, flow cytometry analysis was performed to confirm the presence of surface markers. A high-resolution Apogee A60-Micro-PLUS flow cytometer (Apogee Flow Systems, Hemel Hempstead, UK) was utilized. EV samples were stained with APC-conjugated antibodies against CD63 (clone MEM-259; Thermo Fisher Scientific) and CD81 (clone 5A6; BioLegend, CA, USA) or the corresponding isotype control (IgG1k APC mouse, Miltenyi Biotec, Bergisch Gladbach, Germany).

### 2.5. Transcriptome Analysis

#### 2.5.1. RNA Extraction, RNA-Seq Library Construction, and Sequencing

To the thawed cell pellet kept on ice, 300 μL of TRI Reagent (Thermo Fisher) was added. Total RNA was isolated according to the manufacturer’s protocol. Prior to RNA library construction, samples were additionally purified using Agencourt RNAClean XP (Beckman Coulter, Brea, CA, USA). After library construction using the QuantSeq 3′ mRNA-Seq Library Prep Kit FWD for Illumina, their qualitative (Agilent TapeStation 2200) and quantitative (Qubit, Thermo Fisher Scientific, Waltham, MA, USA) assessment was performed. Quality-checked and normalized libraries were subjected to single-read sequencing with a read length of 2 × 150 bp on a NovaSeq 6000 System (Illumina, San Diego, CA, USA) at The OMRF Clinical Genomics Center (CGC) (Oklahoma City, OK, USA), and only read one was used for analysis. The raw sequencing reads are available in NCBI Sequence Read Archive (SRA) database under BioProject accession number PRJNA1291034.

#### 2.5.2. RNA-Seq Analysis

The provided demultiplexed raw reads were quality-checked using FastQC software [31] and filtered using Flexbar software [32]. High-quality reads were mapped using STAR software to the latest available horse genome (Equ3.0) with ENSEMBL annotation version 107. For read counting, Htseq-count (1.99.2) [33] software was utilized. Subsequent reads normalization, principal component analysis (PCA), hierarchical clustering, and differential expression (DE) analysis were conducted using DESeq2 (v3.16) software [34] implemented on the iDEP.96 (v1.1) platform [35]. Genes were considered differentially expressed if the false discovery rate adjP < 0.05. The DE genes were then analyzed for their function and enrichment in specific Gene Ontology (biological processes, BP) and KEEG pathways categories using iDEP.96 software. Analyzed BP and KEEG annotation categories were considered enriched when the corresponding adjP was lower than 0.05. Corrections for multiple testing were calculated according to the Benjamini–Hochberg method (FDR; false discovery rate) [36].

#### 2.5.3. qPCR

Selected DEGs (*KCNMB2*, *S100A4*, *WNT5A*, *SERPINF1*, *HOXC8*, *IGFBP5*) were tested using RT-qPCR to verify the reliability of the RNA-Seq analysis. For this purpose, cDNA was synthesized using 500 ng of RNA and the High Capacity cDNA Reverse Transcription Kit (Thermo Fisher Scientific), following the manufacturer’s protocol. RT-qPCR was performed with the AmpliQ 5 × HOT EvaGreen^®^ qPCR Mix Plus (ROX) kit (Novazym, Poznan, Poland) and primers designed for mRNA sequences spanning two adjacent exons (Supplementary File S1). Each sample was run in triplicate using Quant Studio 7 Flex (Thermo Fisher Scientific). The relative expression levels of each gene were calculated using the ΔΔCt method [37]. Standardization was performed based on *PPP6R1* gene expression as the internal control [38].

#### 2.5.4. miRNA Isolation, Library Construction, and Sequencing

Total RNA was isolated from EVs using TriReagent and a modified procedure for low amounts. Libraries were prepared using the NEBNext Multiplex Small RNA Library Prep Set for Illumina according to the manufacturer’s protocol [39]. The libraries were sequenced with a read length of 2 × 150 bp on a NovaSeq 6000 system (Illumina, USA) at The OMRF Clinical Genomics Center (CGC) (OK, USA), and only the first 75 bases of read one were used for analysis. The raw sequencing reads are available in NCBI Sequence Read Archive (SRA) database under BioProject accession number PRJNA1289673.

#### 2.5.5. Small RNA-Seq Analysis

The fastq.gz files for each sample were uploaded to the sRNAbench program [34]. The miRBase release 22.1 annotation reference database was selected, and the reads were mapped to the latest available horse genome Equ3.0. For quality control, the filtering method “Use minimum mean quality score, Phred +33 Phred Score Encoding, and 20 Phred Score Threshold” was applied. Parameters were set as follows: seed length for alignment equal to 20, minimum read count equal to 2, minimum read length equal to 15. Differential expression (DE) analysis was performed using sRNAde [40].

## 3. Results

### 3.1. MSCs and Fibroblasts Isolation

Cell lines were obtained from all samples of adipose tissue, bone marrow, and ovaries. Adhesion of MSCs to cell dishes occurred after approximately 48 h, while adhesion of fibroblasts from explants occurred after 5 days. All cells exhibited a fibroblast-like morphology with a central nucleus and abundant cytoplasm.

### 3.2. MSC Differentiation

**Figure 1:**
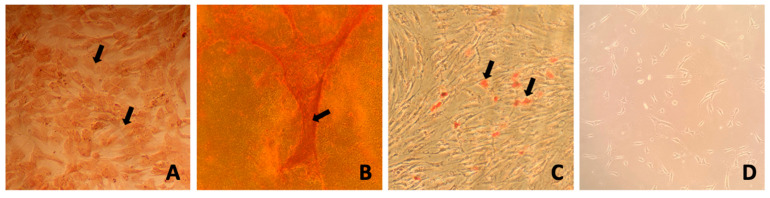
Exemplary results of BM-MSC differentiation into chondrogenic, osteogenic, and adipogenic lineages after three passages. (**A**) Safranin O staining showing cartilage matrix after 21 days in chondrogenic medium. (**B**) Alizarin Red staining of calcium deposits after 21 days in osteogenic medium. (**C**) Oil Red O and hematoxylin staining indicating lipid droplets after 14 days in adipogenic medium. (**D**) MSCs post three passages. Arrows pointing to stained structures: (**A**) intracellular proteoglycans, (**B**) calcified extracellular matrix, and (**C**) lipid droplets.

Trilineage differentiation was performed for MSCs obtained from adipose tissue and bone marrow. During induction, cells exhibited changes in morphology, whereas cells in the negative control (non-induced) did not show any changes. On day 21 of induction, staining with Oil Red O resulted in the staining of lipid droplets formed, Safranin O staining enabled visualization of intracellular proteoglycans, while Alizarin Red allowed for the visualization of calcified extracellular matrix deposits. Hematoxylin used after adipogenic differentiation stained the cytoplasm and cell nuclei blue, allowing for the identification of lipid droplets in differentiated cells. The differentiation results for AT-MSCs were presented in the publication by Sawicki et al., 2023 [29]. Figure 1 shows BM-MSCs after differentiation along with the corresponding controls using specific stains.

### 3.3. Surface Markers of MSC

**Figure 2:**
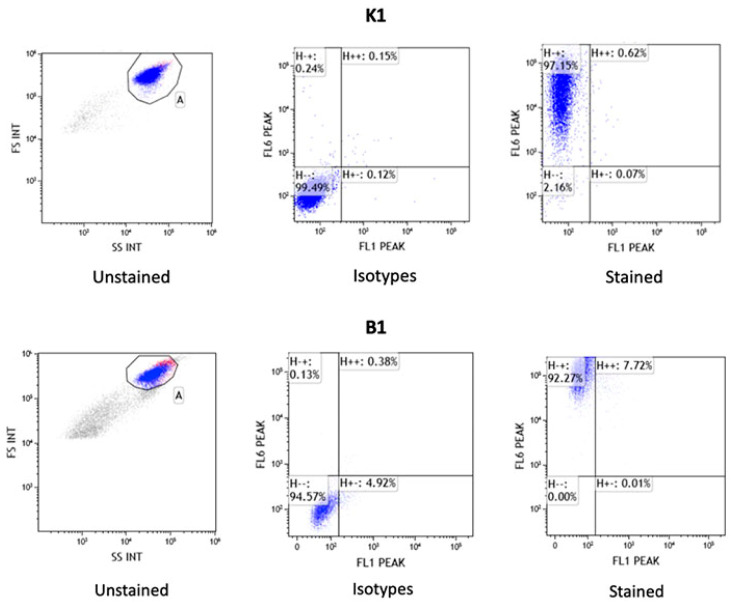
Results of mesenchymal stem cell surface markers analysis by flow cytometry for AT-MSCs (**K1**) and BM-MSCs (**B1**). Graph presents the results for unstained control cells, control cells stained with FITC and APC isotypes, and surface antigens MHC II (FL1 PEAK-FITC) and CD90 (FL6 PEAK-APC).

Among the characterized markers specific to MSCs by the International Society for Cellular Therapy (ISCT), two markers were selected for equine MSCs: CD90 and MHCII. Flow cytometry analysis revealed the presence of the positive surface marker CD90 in approximately 92–99% of all samples of AT-MSCs and BM-MSCs, while the presence of the negative marker MHCII was either undetectable or at low levels around 1–8%. The results of flow cytometry analysis for AT-MSCs and BM-MSCs are presented in Figure 2. The complete data of flow cytometer analysis are shown in Supplementary File S2.

### 3.4. EV Isolation and Characteristics

EVs from all samples were characterized in two ways. (I) Nanoparticle tracking analysis (NTA) and (II) flow cytometry were employed. NTA analysis determined the average size of extracellular vesicles and the concentration of vesicles (particles/mL). The average size of extracellular vesicles obtained from AT-MSCs, BM-MSCs, and fibroblasts was 144.63 ± 1.87 nm, 140.4 ± 2.07 nm, and 147.43 ± 5.57 nm, respectively. The results for each sample are presented in Table 1. To compare the size of extracellular vesicles between the three cell lines, a one-way analysis of variance (ANOVA) was used. The test did not show any significant differences between the analyzed groups (*p* = 0.54). Representative analysis of EVs using NanoSight NS500 for all samples is shown in Supplementary File S3.

**Figure 3:**
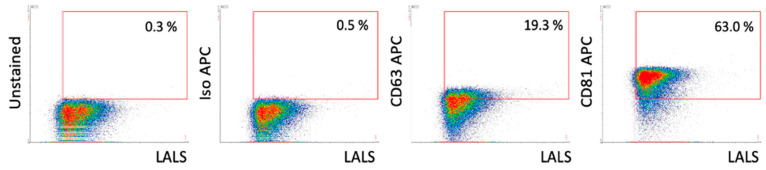
High-resolution flow cytometry analysis of EV samples. Example dot plots of EV-MSC samples from the A60-Micro-PLUS cytometer. The proportions of positive objects for each marker are indicated within the red gating areas. LALS refers to the large-angle light scatter parameter, which correlates with the relative size of the particles analysed.

High-resolution flow cytometry with specific surface markers allowed for phenotypic characterization of EVs in terms of the presence of surface markers CD63 and CD81. An example result for the EV-AT-MSC sample is presented in Figure 3. Results for the remaining samples are shown in Supplementary File S4.

### 3.5. Transcriptome Analysis Results

**Figure 4:**
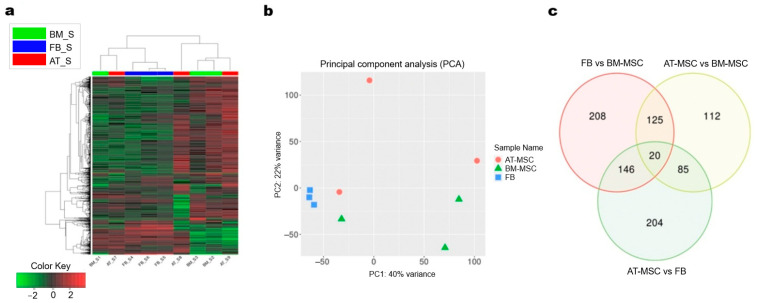
Transcriptomic profiles analysis of adipose-derived MSCs, bone marrow-derived MSCs, and fibroblasts. (**a**) Hierarchical clustering and Tree view visualization of top 1000 most variable differentially expressed genes across the samples (adjP < 0.05). (**b**) PCA plot performed with reads from all expressed genes. BM = bone marrow-derived MSC; FB = fibroblasts; AT = adipose-derived MSC (**c**) Venn diagram of common genes for all comparisons.

Sequencing results of 3′ mRNA technique were obtained for MSCs isolated from bone marrow and adipose tissue, as well as fibroblasts obtained from three different mares. For individual cultures, from 4.8 to 15.5 million reads were generated. On average, 88.3% of filtered reads were successfully mapped to the reference genome, and 6.5% of these reads had multiple mappings. Ultimately, between 59.1% and 67.3% of the mapped reads for individual samples were assigned to genes using the annotation employed for the analysis. Read statistics per sample were shown in Supplementary File S5. Analysis of expression profiles in all analyzed cultures using hierarchical clustering with genes showing the greatest variation between the study groups revealed overall similarity of expression profiles or replicates within fibroblasts group, however, within the two types of MSCs we detected some outliers, for which expression profiles were more similar to fibroblasts (Figure 4a). Similar results were shown using PCA analysis conducted using all expressed genes, which revealed distinct clusters for each cell type, with two MSC samples located near the fibroblast cluster (Figure 4b). The results of the differential analysis are shown in Supplementary File S6. The comparative analysis identified 499 differentially expressed genes (DEGs) between fibroblasts and BM-MSCs, 342 DEGs between AT-MSCs and BM-MSCs, and 455 DEGs between AT-MSCs and fibroblasts.

Comparative analysis between BM-MSCs and AT-MSCs revealed 187 DEGs with increased expression and 155 with lowered expression in AT-MSCs. In the comparison between BM-MSCs and fibroblasts, 332 genes with increased expression and 167 genes with decreased expression were identified in fibroblasts. Meanwhile, the comparison of AT-MSCs with fibroblasts showed 209 DEGs with increased and 246 genes with decreased expression in AT-MSCs.

**Figure 5:**
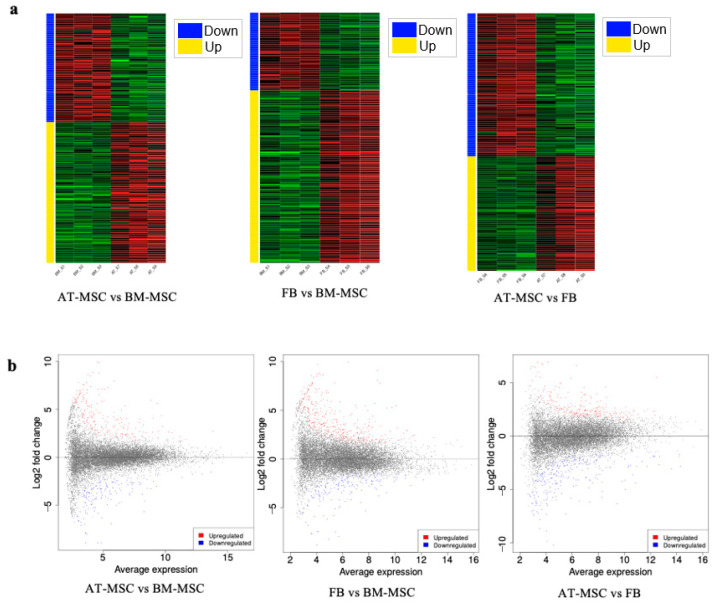
Differential gene expression analysis. (**a**) Heatmaps of down- and upregulated genes between groups. (**b**) MA plots. Significantly differentially expressed genes are marked with the following colors: blue for genes with decreased expression and red or yellow for genes with increased expression.

Furthermore, the comparative analysis of differentially expressed genes revealed 208 uniquely expressed genes for the comparison of fibroblasts with BM-MSC, 204 unique genes for the comparison of AT-MSCs with fibroblasts, and 112 unique genes for AT-MSCs and BM-MSCs. Additionally, 125 DEGs were found to be shared between the fibroblasts vs. BM-MSCs and AT-MSCs vs. BM-MSCs, 146 DEGs were shared between fibroblasts vs. BM-MSCs and AT-MSCs vs. fibroblasts, 112 genes were common to AT-MSCs vs. fibroblasts and AT-MSCs vs. BM-MSCs, and 20 DEGs were shared across all comparisons (Figure 4c). Heatmaps of down- and upregulated genes between the study groups and MA plots representing genes and significantly differentially expressed genes are shown in Figure 5.

### 3.6. Analysis of Differential Genes Between AT-MSCs and BM-MSCs

**Figure 6:**
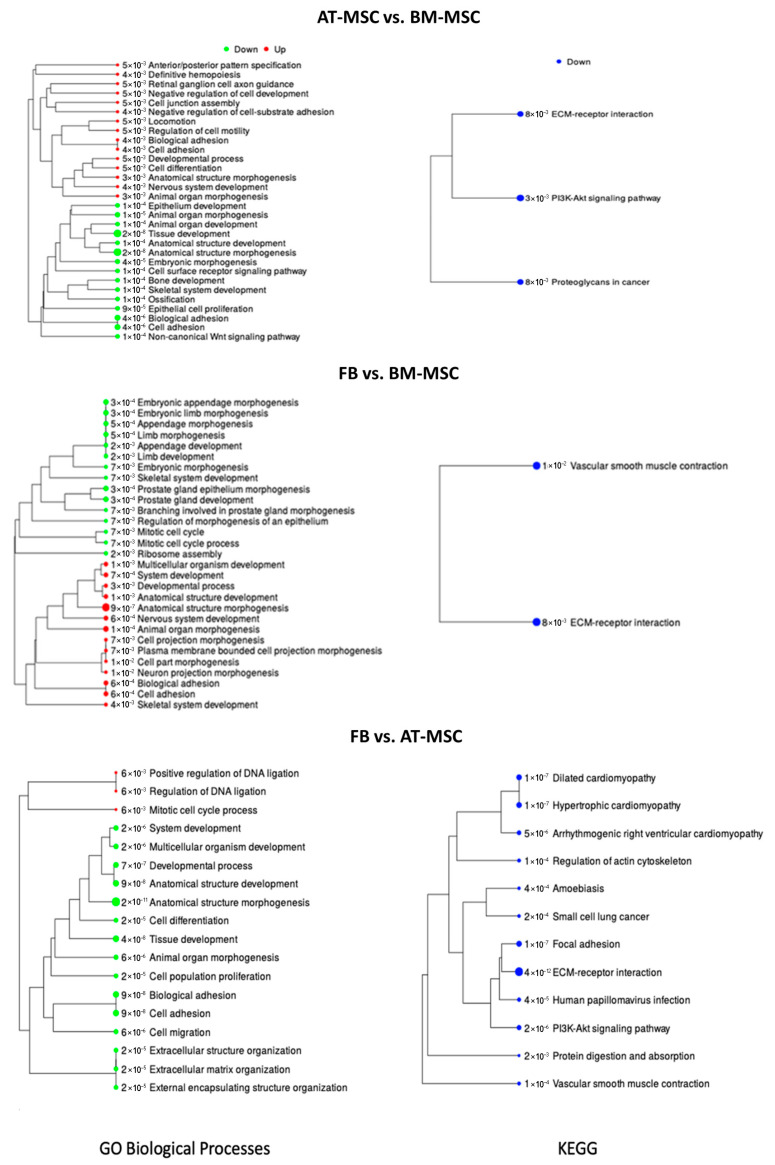
BP and KEEG pathways enrichment tree for transcriptome comparison among AT-MSCs, BM-MSCs, and fibroblasts. The tree represents adjP values, and the size of the dots corresponds to these values.

Genes differentially expressed between AT-MSCs and BM-MSCs were analyzed for related biological processes and KEGG pathways (using the iDEP.96 program).table shows the enrichment tree for each analysis performed. A list of genes with their annotations and statistics for biological processes and KEGG was prepared (Supplementary File S7). When the differentially expressed genes between AT-MSCs and BM-MSCs were analyzed for biological processes, 30 top processes were included, of which 15 were connected with downregulated genes and 15 with upregulated genes in AT-MSCs (Figure 6). The most important biological processes overrepresented by genes with lowered expression were tissue development (GO:0009653; adjP = 2.2 × 10^−8^), embryonic morphogenesis (GO:0048598; adjP = 3.7 × 10^−5^), skeletal system development (GO:0001501; adjP = 1.1 × 10^−4^), ossification (GO:0001503; adjP = 1.2 × 10^−4^), and bone development (GO:0060348; adjP = 1.2 × 10^−4^). Biological processes related to genes with increased expression included nervous system development (GO:0007399; adjP = 3.8 × 10^−3^), definitive hemopoiesis (GO:0060216; adjP = 3.8 × 10^−8^), regulation of cell motility (GO:2000145; adjP = 5.2 × 10^−3^), developmental process (GO:0032502; adjP = 5.2 × 10^−3^), cell junction assembly (GO:0034329; adjP = 5.2 × 10^−3^), locomotion (GO:0040011; adjP = 5.2 × 10^−3^), and cell differentiation (GO:0030154; adjP = 5.2 × 10^−3^). During the analysis, biological processes were also identified that were enriched by both up- and downregulated genes, including cell adhesion (GO:0007155), biological adhesion (GO:0022610), and animal organ morphogenesis (GO:0009887). In the case of KEGG pathways, the analysis allowed the identification of only three pathways related to downregulated genes, including the PI3K-Akt signaling pathway (adjP = 3 × 10^−3^), proteoglycans in cancer (adjP = 8 × 10^−3^), and ECM-receptor interaction (adjP = 8.0 × 10^−3^).

### 3.7. Differences in the Transcription Profile of Fibroblasts and Both Analyzed MSC Types

Figure 6 illustrates the enrichment tree for gene overrepresentation examinations in both BP and KEEG pathways. Each analysis generated a catalog of genes alongside their annotations and statistical data for enrichment of biological processes and KEGG pathways (Supplementary Files S8 and S9).

The examination of differentially expressed genes between fibroblasts and BM-MSCs enabled the identification of 29 overrepresented biological processes. Among enriched BP 15 were downregulated, while 14 were upregulated in fibroblasts (Figure 6). The major biological processes associated with downregulated genes included ribosome assembly (GO:0042255; adjP = 2.3 × 10^−3^), mitotic cell cycle (GO:0000278; adjP = 6.8 × 10^−3^), and embryonic morphogenesis (GO:0048598; adjP = 6.9 × 10^−3^). On the other hand, biological processes associated with upregulated genes encompassed anatomical structure morphogenesis (GO:0009653; adjP = 9.5 × 10^−7^), cell adhesion (GO:0007155; adjP = 5.5 × 10^−4^), nervous system development (GO:0007399; adjP = 5.5 × 10^−4^), biological adhesion (GO:0022610; adjP = 5.5 × 10^−4^), multicellular organism development (GO:0007275; adjP = 9.7 × 10^−4^), anatomical structure development (GO:0048856; adjP = 9.7 × 10^−4^), and cell projection morphogenesis (GO:0048858; adjP = 6.8 × 10^−3^). Further analysis led to the identification of two KEGG pathways exclusively associated with upregulated genes: ECM-receptor interaction (adjP = 8.0 × 10^−3^) and vascular smooth muscle contraction (adjP = 1.0 × 10^−2^).

To evaluate the functions of DEGs between AT-MSCs and fibroblasts, a similar approach was employed, resulting in the preparation of a list of genes with their respective annotations and statistics for biological processes and KEGG pathways (Supplementary File S9). Figure 6 depicts the enrichment tree for gene overrepresentation tests in BP and KEGG pathways. Analysis identified 18 enriched biological processes, of which 15 were connected with downregulated genes in AT-MSCs, and 3 were associated with upregulated genes in AT-MSCs (Figure 5). The biological processes enriched by upregulated genes included the mitotic cell cycle process (GO:1903047; adjP = 5.7 × 10^−3^), regulation of DNA ligation (GO:0051105; adjP = 5.7 × 10^−3^), and positive regulation of DNA ligation (GO:0051106; adjP = 5.7 × 10^−3^). Notably, the most significant biological processes enriched by downregulated genes comprised tissue development (GO:0009888; adjP = 3.6 × 10^−8^), cell adhesion (GO:0007155; adjP = 9.0 × 10^−8^), biological adhesion (GO:0022610; adjP = 9.0 × 10^−8^), anatomical structure development (GO:0048856; adjP = 9.0 × 10^−8^), multicellular organism development (GO:0007275; adjP = 1.8 × 10^−6^), and cell differentiation (GO:0030154; adjP = 1.8 × 10^−5^). Additionally, 12 KEGG pathways, enriched exclusively with downregulated genes, were identified, with notable pathways including ECM-receptor interaction (adjP = 4 × 10^−12^), focal adhesion (adjP = 1 × 10^−7^), hypertrophic cardiomyopathy (adjP = 1 × 10^−7^), PI3K-Akt signaling pathway (adjP = 2 × 10^−6^), small cell lung cancer (adjP = 2 × 10^−4^), and protein digestion and absorption (adjP = 2 × 10^−3^).

### 3.8. PCR Validation of NGS Data

To confirm the reliability of the results obtained using the RNA-seq method, six genes (*KCNMB2*, *S100A4*, *WNT5A*, *SERPINF1*, *HOXC8*, *IGFBP5*), selected from the 20 common to all cell line comparisons, were examined using qPCR (Supplementary File S10). The analysis of the obtained results showed that gene expression measured by RNA-seq and qPCR was highly correlated, with a correlation coefficient (r) ranging from 0.688 to 1 for individual genes and a *p*-value < 0.05 for the correlation coefficient. The results obtained from the qPCR and NGS analyses and summary of the correlation analysis of gene expression from both laboratory methods, for samples and mean values for groups, as well as the fold-change parameter for individual genes are presented in Supplementary File S10.

### 3.9. miRNA Analysis

**Figure 7:**
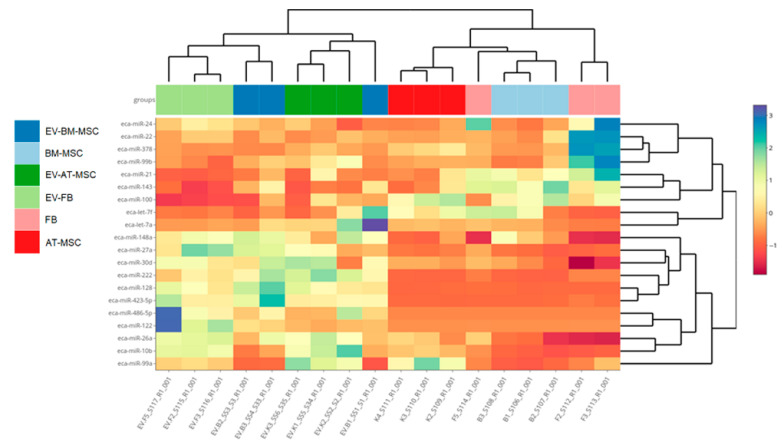
Hierarchical clustering of expression profiles of all studied cultures based on genes with the highest differentiation among the groups. B1, B2, B3—samples of BM-MSCs; F1, F2, F3—fibroblasts; K1, K2, K3—AT-MSCs; EV-K1, EV-K2, EV-K3—AT-MSC-derived EVs; EV-B1, EV-B2, EV-B3—BM-MSC-derived EVs; EV-F1, EV-F2, EV-F3—fibroblast-derived EVs.

The investigation concerned the differential expression analysis of miRNAs between individual cell types and the extracellular vesicles they secreted. The difference in miRNA expression profile clustering was especially visible for miRNA derived from extracellular vesicles and miRNA derived from cells (Figure 7). The number of mature miRNAs, hairpin miRNAs, and novel miRNAs for cells from each source are presented in Table 2. The analysis identified two novel miRNAs for each cell type. Identified novel miRNAs in *Equus caballus* showed perfect sequence identity with bta-miR-6119-5p from *Bos taurus* and mmu-miR-1247-3p from *Mus musculus*. In this manuscript, we refer to this miRNA as eca-miR-6119-5p and eca-miR-1247-3p, respectively, pending official miRBase submission and annotation. A summary of the miRNA content in all cell types and extracellular vesicles was also prepared, allowing for the identification of unique miRNA for each structure (Supplementary File S11).

Subsequently, an analysis was conducted to identify upregulated and downregulated miRNA by comparing different cells (Table 3) and extracellular vesicles (EVs) (Table 4). The results of the differential miRNA expression analysis are presented in the Supplementary Materials (Supplementary File S12). For the comparison of BM-MSCs and AT-MSCs, 39 downregulated miRNA and 45 upregulated miRNA were obtained. For the comparison of fibroblasts and AT-MSCs, 50 downregulated miRNA and 10 upregulated miRNA were obtained. For the comparison of fibroblasts and BM-MSCs, 47 downregulated miRNA and 10 upregulated miRNA were obtained (Table 4). Differential miRNA expression analysis was also performed for EV-derived miRNAs collected from these cells (Supplementary File S13). For the comparison of EV-derived BM-MSCs and EV-derived AT-MSCs, two downregulated miRNA and seven upregulated miRNA were obtained. For the comparison of EV-derived fibroblasts and EV-derived AT-MSCs, 37 downregulated miRNA and 21 upregulated miRNA were obtained. For the comparison of EV-derived fibroblasts and EV-derived BM-MSCs, 30 downregulated miRNA and 15 upregulated miRNA were obtained. Results of the differential expression analysis between individual cell types and the extracellular vesicles they secreted are presented in the Supplementary Materials (Supplementary File S14).

**Figure 8:**
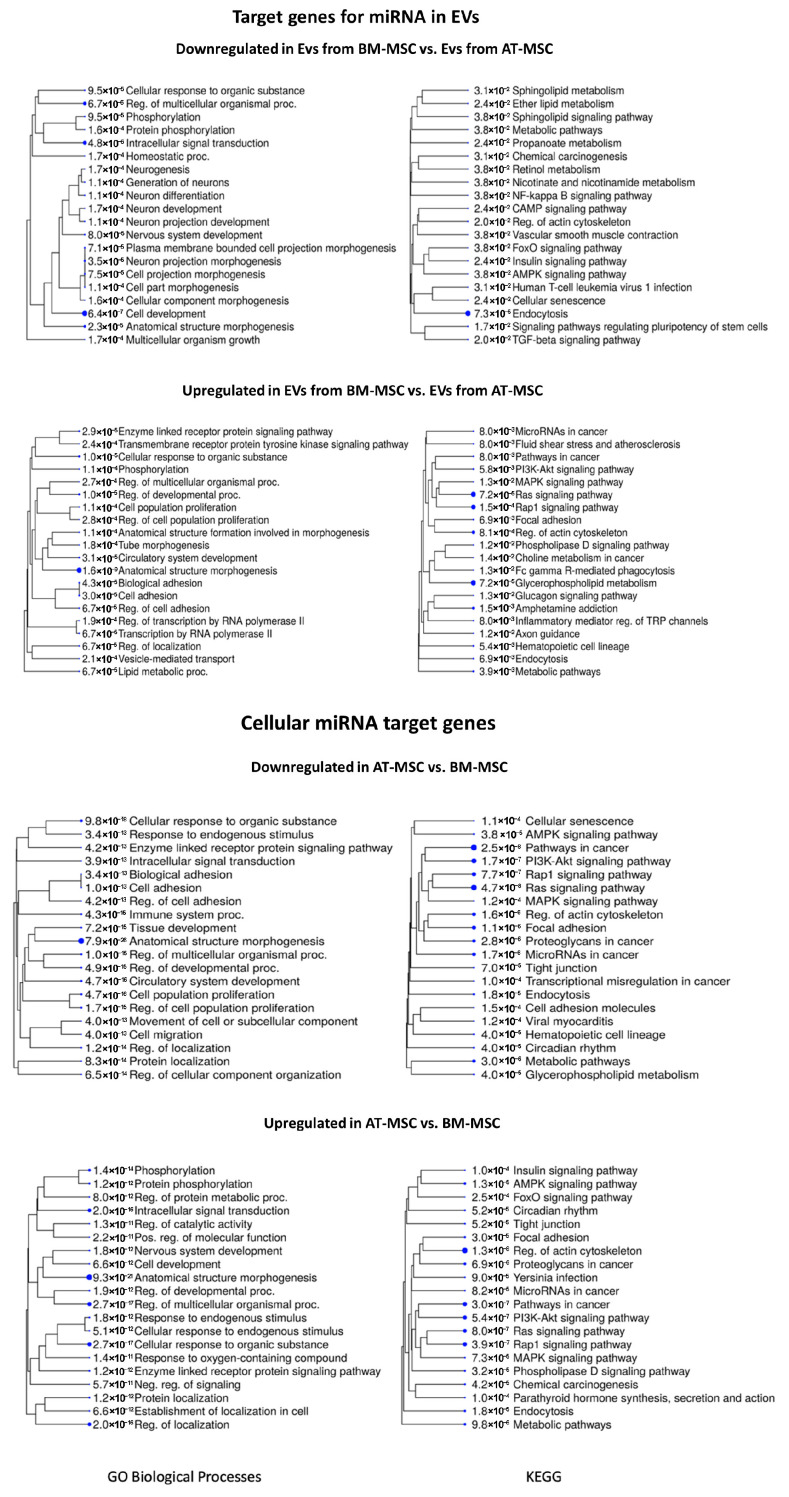
The 20 most significant biological processes and KEGG pathways for target genes associated with downregulated and upregulated miRNAs in EVs and cells derived from BM-MSCs compared to AT-MSCs. The tree represents adjP values, and the size of the dots corresponds to these values.

Figure 8 presents the biological processes and KEGG pathways associated with target genes for miRNA in BM-MSC-derived EVs compared to AT-MSC-derived EVs. For the comparison of EVs derived from BM-MSCs to EVs derived from AT-MSCs, the most statistically significant identified downregulated biological processes were cell development (GO:0048468; adjP = 6.4 × 10^−7^), intracellular signal transduction (GO:0035556; adjP = 4.8 × 10^−6^), and regulation of multicellular organismal process (GO:0051239; adjP = 6.7 × 10^−5^), while for the KEGG pathways, they were endocytosis (adjP = 7.3 × 10^−5^), signaling pathways regulating pluripotency of stem cells (adjP = 1.7 × 10^−2^), and TGF-beta signaling pathway (adjP = 2.0 × 10^−2^). Among the upregulated biological processes, the most statistically significant were anatomical structure morphogenesis (GO:0009653; adjP = 1.6 × 10^−9^), regulation of developmental process (GO:0050793; adjP = 1.0 × 10^−5^), cellular response to organic substance (GO:0071310; adjP = 1.0 × 10^−5^), while the most enriched KEGG pathways included: glycerophospholipid metabolism (adjP = 7.2 × 10^−5^), Ras signaling pathway (adjP = 7.2 × 10^−5^), and rap1 signaling pathway (adjP = 1.5 × 10^−4^).

Figure 8 further summarize the processes and pathways for comparison between AT-MSCs and BM-MSCs. For the comparison of AT-MSCs to BM-MSCs, the most significant identified downregulated biological processes were anatomical structure morphogenesis (GO:0009653; adjP = 7.9 × 10^−26^), cellular response to organic substance (GO:0071310; adjP = 9.8 × 10^−18^), and cell population proliferation (GO:0008283; adjP = 4.7 × 10^−16^), while for the KEGG pathways, they were pathways in cancer (adjP = 2.5 × 10^−8^), Ras signaling pathway (adjP = 4.7 × 10^−8^), and PI3K-Akt signaling pathway (adjP = 1.7 × 10^−7^). Among the upregulated biological processes, the most statistically significant were anatomical structure morphogenesis (GO:0009653; adjP = 9.3 × 10^−21^), regulation of multicellular organismal process (GO:0051239; adjP = 2.7 × 10^−17^), and cellular response to organic substance (GO:0071310; adjP = 2.7 × 10^−17^), while the most enriched KEGG pathways included regulation of actin cytoskeleton (adjP = 1.3 × 10^−8^), pathways in cancer (adjP = 3.0 × 10^−7^), and rap1 signaling pathway (adjP = 3.9 × 10^−7^).

The Supplementary File S14 presents a comparison of biological processes and KEGG pathways between fibroblasts and stem cells, as well as between the extracellular vesicles secreted by these cells. Results of the most significant biological processes and KEGG pathways for target genes associated with downregulated and upregulated miRNAs in cells and extracellular vesicles are presented in Supplementary File S15. Additionally, an analysis of target genes for miRNAs is provided in Supplementary File S16. Supplementary Files S17 and S18 show the target genes of miRNAs for biological processes and KEGG pathways, respectively.

## 4. Discussion

Stem cell transplantation represents an attractive technique applicable in tissue engineering and regenerative medicine. The versatility of stem cells prompts research into the mechanisms of their action, largely controlled by factors regulating gene expression. However, to fully understand the transcriptional profile and its variability across different MSC types, comprehensive comparative studies at the transcriptome level are necessary. The outcome of these studies is a comprehensive transcriptional characterization of various MSC types, providing insights into metabolic pathways that differentiate individual types and inferring their suitability for various applications. Differences between MSC types may also manifest in the characteristics and content of secreted EVs, which mediate important MSC effects [11].

In this study, MSCs were isolated from the bone marrow and adipose tissue of horses, and fibroblasts were isolated from the horse ovary. Mesenchymal stem cells were identified according to the criteria established by ISCT [7,41]. Extracellular vesicles were also isolated from the cells, which were identified according to the latest MISEV2018 guidelines [14].

In the transcriptomic analyses, hierarchical clustering of expression profiles was conducted, revealing significant differences between the analyzed cell groups, largely corresponding to their tissue origins. It is worth noting that one biological sample of MSCs from adipose tissue and one biological sample of MSCs from bone marrow exhibited an expression profile similar to fibroblasts. It is possible that this phenomenon results from the presence of cell population contamination that was not effectively separated during the cultivation process. A similar effect has been previously described in the literature in the context of using mixed cell cultures in cattle [42] and horses [43,44]. Nonetheless, the remaining two cultures showed expression profiles different from fibroblasts, suggesting a high level of homogeneity.

The studies conducted by Uder C., et al., 2018 [45] and by Harman R. M., et al., 2020 [6] showed similarities between human and equine MSCs. This allows for the interpretation of the obtained results by referring to studies conducted on human MSCs. MSCs isolated from different tissue sources exhibit distinct phenotypic characteristics and possess different therapeutic abilities [45]. In our study, cells exhibited clear differences in gene expression, which can be attributed to their tissue origin.

The comparative analysis of the two studied types of MSCs revealed differences in the regulation of biological processes related to tissue development, embryonic morphogenesis, and the development of the skeletal system and bones. In the case of AT-MSCs, downregulated genes relative to BM-MSCs were observed and were associated with the regulation of bone homeostasis, stiffness, stabilization of the extracellular matrix, and bone development. This may reflect the origin of BM-MSCs from the skeletal system. Available studies have shown that different sources of MSCs affect their biological properties and regenerative potential [46]. Studies conducted on humans have demonstrated that BM-MSCs are involved in membranous ossification and the formation of ectopic hematopoietic niches [47,48]. It has also been shown that BM-MSCs differentiate better into ectopic bone compared to MSCs derived from adipose tissue [49].

Analyzing changes in gene expression related to the process of bone development, it was found that one of the important genes with decreased expression in MSCs derived from adipose tissue compared to BM-MSCs was the *WNT5A* gene, which belongs to the *WNT* gene family responsible for regulating cell fate during embryogenesis [50]. *WNT5A* (Wnt family member 5A) may play a significant role in the development of periodontal tissues and the regulation of bone homeostasis [51]. Studies also suggest that *WNT5A* positively influences the regeneration processes of the periodontal complex [50]. The *WNT* signaling pathway plays a crucial role in the development and homeostasis of the skeletal system by promoting the proliferation, differentiation, and maturation of osteoblasts [51,52].

Another important gene associated with bone development is the *sFRP4* gene (secreted frizzled-related protein 4), which is characterized by decreased expression in AT-MSCs compared to BM-MSCs. As a negative regulator of the *Wnt* signaling pathway, it affects bone mass [53]. Modulation of *Wnt* signaling by *sFRP4* plays a significant role in the development of the skeletal system after birth and in maintaining bone in adults [54]. This gene exhibits increased expression during early embryonic stages [55] and plays an important role in MSCs, participating in autocrine and paracrine signaling mechanisms [56].

The analysis also revealed increased expression of the *DLX5* and *DLX6* genes in BM-MSCs compared to AT-MSCs and fibroblasts. These genes are dynamic regulators of mammalian development [57], absolutely essential for the proper development of the skull and skeletal system, and exhibit overlapping functions in all tissues where their expression occurs [57]. These genes are also crucial for the development of embryonic organs [58]. In the analysis of processes related to embryonic development, the *TGM2* gene (transglutaminase 2) was identified with decreased expression in AT-MSCs compared to BM-MSCs. This gene is crucial for tissue development, participating in various processes such as cell growth and differentiation as well as the stabilization of the extracellular matrix [59].

Significant differences were also observed in the expression of genes from the *LOX* family (lysyl oxidase), which are copper-dependent amine oxidases critical for the formation of covalent cross-links in collagen and elastin, contributing to the strengthening of the extracellular matrix [60]. The importance of the *LOXL3* gene has been confirmed in studies using a zebrafish model, where the absence of *LOXL3B* led to developmental defects in the facial skeleton [61]. Studies on *LOXL3*-deficient mice (*Loxl3*−/−) demonstrated severe developmental abnormalities, such as cleft palate, shortened mandible, and spinal cord deformities, highlighting the critical role of *LOXL3* in embryonic survival and proper development [62].

The results of these studies and the transcriptomic analysis suggest that BM-MSCs have a significant differential regulation of genes important for early organism development, particularly in the process of bone development, compared to AT-MSCs.

On the other hand, AT-MSCs exhibited increased expression of the *GATA5* gene, which, along with the *GATA4* and *GATA6* genes, is involved in the development of organs such as the heart and intestine [63], suggesting that AT-MSCs demonstrate a better plasticity towards the construction of organs derived from the endoderm compared to BM-MSC. In early embryonic development, *GATA5* assists in generating a sufficient number of precursor cells for cardiac muscle, allowing them to differentiate into mature cardiomyocytes and also regulating other genes essential for heart development [64]. 

Stem cells from both sources also differed in the expression levels of genes related to cell adhesion. One such gene was the transcription factor *GATA2*, which exhibited increased expression in BM-MSCs compared to AT-MSCs and is responsible for regulating genes involved in cell adhesion (i.e., it reduces the expression of adhesion molecules–*ITGA11* and *ITGB3*) and chemotaxis in BM-MSCs [65]. Additionally, the transcription factor *GATA2* is essential for the functioning of hematopoietic stem cells and other blood lineages, suggesting that it may help maintain bone marrow mesenchymal stem cells in an immature state while also contributing to their differentiation [66].

Another gene with increased expression in BM-MSCs compared to AT-MSCs was the *FAP* gene, which is expressed only in mesodermal stem cells of the bone marrow in adult tissues [67]. Previous studies in humans have shown that *FAP* peptidase activity is not significantly important for the migration of BM-MSCs; however, it regulates cell adhesion by modulating RhoA GTPase activity, proteins that play key roles in controlling essential cellular processes [68].

Differences were also observed between AT-MSCs and BM-MSCs in the expression of genes associated with the *PI3K*/*AKT* signaling pathway, which plays a crucial role in many cellular functions such as cell proliferation, cell transformation, cell cycle progression, survival, cellular metabolism, paracrine functions, and angiogenesis [69,70,71].

It has also been demonstrated that medium-containing stem cells in rats attenuated cardiac muscle injury during reperfusion, and the cardioprotective effect was due to the activation of the *PI3K*/*AKT* pathway through paracrine factors [72]. These observations suggest a significant role for the *PI3K*/*AKT* pathway in the paracrine function of BM-MSCs.

In 2008, Covas et al. [73] found that MSCs, pericytes, and fibroblasts exhibited a similar gene expression pattern, but their immunophenotype and differentiation experiments led to the conclusion that fibroblasts are differentiated cells with limited differentiation potential. On the other hand, differences in the expression profiles of certain genes have been effectively used to distinguish fibroblasts from MSCs [74].

*FN1* (fibronectin 1) was one of the genes with increased expression in BM-MSCs compared to AT-MSCs and fibroblasts, as confirmed in other studies on humans [75]. This glycoprotein plays a key role in the organization and composition of the extracellular matrix and in intercellular adhesion sites [76]. Since MSCs can be isolated by adhesion growth on culture vessels coated with fibronectin [77], it seems that this interaction between cells and the matrix may have particular significance for MSC development.

An important gene that exhibited higher expression in fibroblasts compared to MSCs from both sources was the *CCN3* gene (cellular communication network factor 3). This gene is involved in the proliferation and apoptosis of fibroblasts as well as in the synthesis of extracellular matrix proteins [78], suggesting an important role in scar formation. This may indicate a greater potential of fibroblasts in skin wound healing compared to MSCs.

The miRNA sequencing conducted in this study on cells from different sources and the EVs secreted by these cells supplemented and confirmed the results of the mRNA analysis, showing that the cells have distinct transcriptional profiles that likely reflect the nature of the niche from which they originate. Additionally, the comparative analysis of miRNA content in EVs and cells provided a basis for examining the mechanism of RNA secretion into EVs. The miRNA content was comparable across all types of studied cells and their secreted EVs. However, certain types of miRNAs were observed only in specific cell types or secreted vesicles, confirming the specific organization of miRNA content in EVs prior to their secretion.

Previous studies have shown that microRNA in EVs derived from MSCs play a significant role in the negative regulation of apoptosis, fibrosis, inflammatory conditions, and oxidative stress, as well as in promoting proliferation and angiogenesis [79]. Research on miRNAs obtained from EVs has practical implications in cell therapy, as the molecular cargos of vesicles derived from stem cells can influence numerous processes occurring in the microenvironment, leading to significant changes in cell phenotypes [80]. Therefore, a detailed identification of EV components would provide a better understanding of the therapeutic effects of these structures in animals.

Analysis conducted in pigs revealed increased expression of miR-218, which regulates osteogenic differentiation [81], and miR-148a, which regulates the process of angiogenesis [82]. In the current studies, BM-MSCs exhibited elevated expression of miR-148a compared to AT-MSCs, suggesting that BM-MSCs regulate the angiogenesis process. Conversely, increased expression of miR-218 was observed in both AT-MSCs and BM-MSCs compared to fibroblasts. Previous reports suggested that the relationship between miR-218 and the Wnt/β-catenin signaling pathway promotes osteogenic differentiation of stem cells derived from human adipose tissue [71]. Therefore, miR-218 may play a significant role in regulating the osteogenic differentiation of stem cells from both adipose tissue and bone marrow.

Extracellular vesicle (EV)-derived miRNAs hold significant translational potential in clinical settings due to their stability in biological fluids and their ability to reflect the physiological and pathological state of their cells of origin. As minimally invasive biomarkers, EV-miRNAs can provide valuable diagnostic and prognostic information across a wide range of diseases, including cancers [83]. Moreover, EVs serve as natural carriers capable of delivering therapeutic miRNAs or other molecular cargo to target cells, offering a promising platform for novel treatment strategies [84]. The ability to engineer EVs for targeted delivery further enhances their appeal as therapeutic vesicles, potentially enabling precise modulation of gene expression with reduced off-target effects. Thus, continued research into EV-derived miRNAs may open new avenues for both disease monitoring and innovative therapies in personalized medicine.

This study has several limitations that should be acknowledged. First, the use of post-mortem tissue may introduce biological variability that could affect interpretation. Second, the small sample size (*n* = 3 per group) limits statistical power and generalizability. Third, the lack of functional assays prevents conclusions about the biological relevance of the identified miRNAs. Furthermore, the use of fibroblasts isolated from ovarian stroma might introduce some further results uncertainty. It should be considered that fibroblasts isolated from the ovarian stroma have certain distinctive features due to their anatomical origin and biological function, consistent with the well-documented heterogeneity and “tissue memory” of fibroblasts [85]. While they share typical characteristics of fibroblasts, such as a spindle-shaped morphology and adherence to plastic, they differ in gene expression, responsiveness to hormones, and their role in the ovarian microenvironment [86,87]. Ovarian fibroblasts may also have the potential to differentiate into thecal cells or other hormonally active stromal elements, a potential not seen in fibroblasts from tissues such as skin or lung [88]. Nonetheless, they share many core characteristics with fibroblasts derived from other tissues, such as skin, lung, or adipose tissue. These similarities include their spindle-shaped morphology, adherence to plastic surfaces in culture, and expression of common mesenchymal markers, such as vimentin and fibronectin [89]. Like other fibroblasts, they are capable of producing and remodeling extracellular matrix (ECM) components, particularly various types of collagen [90]. They also have a similar proliferative capacity in vitro and contribute to tissue structure, mechanical integrity, and wound healing [91]. Functionally, they respond to general signals like inflammatory cytokines (e.g., IL-1β, TNF-α) and growth factors (e.g., TGF-β, FGF), which regulate fibroblast behavior across tissues, including the ovarian stroma [92]. These shared properties make fibroblasts (regardless of origin) fundamentally important for maintaining connective tissue homeostasis, and a good point of reference in transcriptomics studies on other cell types. Despite all these limitations, the findings highlight the potential translational value of EV-derived miRNAs. Given their stability in body fluids and capacity to reflect tissue states, EV-miRNAs hold promise as non-invasive biomarkers and therapeutic vesicles. Further studies involving lager and more representative sample populations, combined with functional validation, are warranted to explore their clinical utility.

In summary, the analysis of transcriptomic profiles performed in this study revealed significant differences between the studied cells, largely reflecting their type and tissue of origin. The results identified genes with altered expression across different MSC types, associated with key biological processes essential for stem cell function, such as e.g., adhesion and differentiation. miRNA sequencing of cells from various sources, as well as of the EVs they secrete, demonstrated distinct expression profiles likely reflecting the characteristics of their tissue-specific niches. The EVs exhibited unique miRNA signatures, suggesting selective packaging and secretion mechanisms. Moreover, the predicted target genes of the identified miRNAs differed between the cells and their EVs, indicating potentially distinct roles in the regulation of biological processes in recipient cells.

## Figures and Tables

**Table 1:** The average size and concentration of extracellular vesicles along with the standard deviation in the NTA analysis.

Sample	Source	Mean ± Stand. Error (nm)	Concentration (particles/mL)
K1	AT-MSC	149.8 ± 2.2	1.47 × 10^13^ ± 8.81 × 10^11^
K2	AT-MSC	138.7 ± 2.0	9.58 × 10^12^ ± 3.91 × 10^11^
K3	AT-MSC	145.4 ± 1.4	5.72 × 10^12^ ± 2.27 × 10^11^
B1	BM-MSC	133.7 ± 2.3	4.79 × 10^12^ ± 2.04 × 10^11^
B2	BM-MSC	133.7 ± 1.1	3.87 × 10^12^ ± 8.53 × 10^10^
B3	BM-MSC	153.8 ± 2.8	8.89 × 10^12^ ± 4.51 × 10^11^
F1	FB	133.7 ± 7.2	8.67 × 10^11^ ± 5.67 × 10^10^
F2	FB	156.2 ± 7.4	7.47 × 10^11^ ± 5.59 × 10^10^
F3	FB	152.4 ± 2.1	1.12 × 10^12^ ± 6.95 × 10^10^

**Table 2:** The number of detected miRNA types for cells and EVs from each source.

	BM-MSC	AT-MSC	Fibroblast	EV-BM-MSC	EV-AT-MSC	EV-Fibroblast
Mature miRNA	209	251	230	138	145	206
Harpin miRNA	256	234	192	83	110	154
Novel miRNA	eca-miR-6119-5p, new-mir-novel1	eca-miR-1247-5p, new-mir-novel1	eca-miR-1247-3p, new-mir-novel1	0	0	0

**Table 3:** MiRNAs with differential abundance in cells and EVs between groups (adjP < 0.05).

Comparison	Downregulated	Upregulated
BM-MSCs vs. AT-MSCs	eca-miR-501-5p, eca-miR-532-5p, eca-miR-10b, eca-let-7c, eca-miR-99a, eca-miR-758, eca-miR-543, eca-miR-409-5p, eca-miR-493b, eca-miR-615-3p, eca-miR-494, eca-miR-495, eca-miR-382, eca-miR-136, eca-miR-370, eca-miR-134, eca-miR-3958, eca-miR-10a, eca-miR-381, eca-miR-411, eca-miR-409-3p, eca-miR-379, eca-miR-127, eca-miR-196a	eca-miR-148a, eca-miR-145, eca-miR-7, eca-miR-143, eca-miR-342-3p, eca-miR-133a, eca-miR-181a, eca-miR-181b, eca-miR-486-5p, eca-miR-224
AT-MSCs vs. Fibroblasts	eca-miR-423-5p, eca-miR-7, eca-miR-143, eca-miR-222, eca-miR-99b, eca-miR-181b, eca-miR-21, eca-miR-28-3p, eca-miR-378, eca-miR-1307, eca-miR-181a, eca-miR-24, eca-miR-193a-5p, eca-miR-328, eca-miR-411, eca-miR-193b, eca-miR-23b, eca-miR-145, eca-miR-22, eca-miR-744, eca-miR-342-3p, eca-miR-129a-5p, eca-miR-129b-5p, eca-miR-342-5p, eca-miR-494, eca-miR-3548, eca-miR-133a, eca-miR-8995, eca-miR-409-5p, eca-miR-543, eca-miR-487b, eca-miR-136, eca-miR-379, eca-miR-758, eca-miR-485-3p, eca-miR-495, eca-miR-122, eca-miR-127, eca-miR-299, eca-miR-381, eca-miR-485-5p, eca-miR-409-3p, eca-miR-493b, eca-miR-382, eca-miR-323-3p, eca-miR-3958, eca-miR-134, eca-miR-370, eca-miR-432, eca-miR-431	eca-miR-199a-5p, eca-miR-101, eca-miR-10b, eca-miR-199b-5p, eca-miR-99a, eca-miR-30c, eca-miR-218, eca-miR-148a, eca-miR-196a, eca-miR-196b
BM-MSCs vs. Fibroblasts	eca-miR-532-5p, eca-miR-501-5p, eca-miR-191a, eca-miR-99a, eca-miR-99b, eca-miR-222, eca-miR-502-3p, eca-miR-744, eca-miR-193a-5p, eca-miR-24, eca-let-7c, eca-miR-22, eca-miR-411, eca-miR-329b, eca-miR-136, eca-miR-493b, eca-miR-495, eca-miR-432, eca-miR-382, eca-miR-379, eca-miR-370, eca-miR-134, eca-miR-127, eca-miR-381, eca-miR-3958, eca-miR-409-3p, eca-miR-378, eca-miR-132, eca-miR-8995, eca-miR-196a, eca-miR-122, eca-miR-656, eca-miR-615-3p, eca-miR-299, eca-miR-10a, eca-miR-3959, eca-miR-187, eca-miR-615-5p, eca-miR-487b, eca-miR-323-3p, eca-miR-485-3p, eca-miR-543, eca-miR-409-5p, eca-miR-431, eca-miR-494, eca-miR-485-5p, eca-miR-758	eca-miR-199a-5p, eca-miR-214, eca-miR-199b-5p, eca-miR-181b, eca-miR-30c, eca-miR-101, eca-miR-340-5p, eca-miR-218, eca-miR-148a, eca-miR-196b

**Table 4:** MiRNAs with differential abundace in EVs between groups (adjP < 0.05).

Comparison	Downregulated	Upregulated
AT-MSC-derived EVs vs. BM-MSC-derived EVs	eca-miR-143, eca-miR-181b	eca-miR-532-5p, eca-miR-10b, eca-let-7c, eca-miR-99a, eca-miR-615-3p, eca-miR-10a, eca-miR-363
AT-MSC-derived EVs vs. Fibroblast-derived EVs	eca-miR-29a, eca-miR-25, eca-miR-181a, eca-miR-191a, eca-miR-30c, eca-miR-27b, eca-miR-186, eca-miR-30e, eca-miR-192, eca-miR-23b, eca-miR-381, eca-miR-23a, eca-miR-181b, eca-miR-101, eca-miR-495, eca-miR-122, eca-miR-409-3p, eca-miR-379, eca-miR-451, eca-miR-543, eca-miR-505, eca-miR-224, eca-miR-382, eca-miR-656, eca-miR-1839, eca-miR-342-3p, eca-miR-194, eca-miR-370, eca-miR-136, eca-miR-411, eca-miR-126-3p, eca-miR-106b, eca-miR-485-5p, eca-miR-361-5p, eca-miR-494, eca-miR-139-5p, eca-miR-3958	eca-miR-99b, eca-miR-99a, eca-miR-222, eca-miR-125a-5p, eca-miR-532-5p, eca-miR-21, eca-miR-7, eca-miR-100, eca-miR-125b-5p, eca-miR-199a-5p, eca-let-7a, eca-miR-28-5p, eca-miR-196b, eca-miR-1271a, eca-miR-199b-5p, eca-let-7c, eca-let-7e, eca-miR-671-3p, eca-miR-193b, eca-miR-29b, eca-miR-151-5p
BM-MSC-derived EVs vs. Fibroblast-derived EVs	eca-miR-10b, eca-miR-186, eca-miR-101, eca-miR-99a, eca-miR-122, eca-miR-192, eca-miR-23b, eca-miR-191a, eca-miR-30e, eca-miR-451, eca-miR-146b-5p, eca-miR-381, eca-miR-127, eca-miR-494, eca-miR-30c, eca-miR-3958, eca-miR-126-3p, eca-miR-139-5p, eca-miR-140-3p, eca-miR-379, eca-miR-543, eca-miR-409-3p, eca-miR-615-3p, eca-miR-382, eca-miR-485-5p, eca-miR-361-5p, eca-miR-411, eca-miR-370, eca-miR-10a, eca-miR-495	eca-miR-222, eca-miR-100, eca-miR-125b-5p, eca-miR-199a-3p, eca-miR-199a-5p, eca-miR-199b-3p, eca-miR-328, eca-let-7a, eca-miR-143, eca-miR-199b-5p, eca-let-7e, eca-miR-214, eca-miR-28-5p, eca-miR-671-3p, eca-miR-151-5p

## Data Availability

Raw sequencing data are submitted to the public Sequence Read Archive (SRA) database under accession numbers PRJNA1291034 and PRJNA1289673.

## Supplementary Materials

The following supporting information can be downloaded at: https://www.mdpi.com/article/10.3390/genes16080936/s1, Supplementary File S1: Primers used for qPCR gene expression validation; Supplementary File S2: Results of analysis of mesenchymal stem cell surface markers using flow cytometry; Supplementary File S3: Results of nanoparticle tracking analysis (NTA) for the isolated EVs; Supplementary File S4: Results of high-resolution flow cytometry of EV samples; Supplementary File S5: Sequencing read statistics; Supplementary File S6: Results of differential mRNA analysis; Supplementary File S7: Results of gene overrepresentation tests in GO biological processes and KEGG pathways for DEGs from the AT vs. BM comparison; Supplementary File S8: Results of gene overrepresentation tests in GO biological processes and KEGG pathways for DEGs from the FB vs. BM comparison; Supplementary File S9: Results of gene overrepresentation tests in GO biological processes and KEGG pathways for DEGs from the FB vs. AT comparison; Supplementary File S10: List of the 20 common genes for each cell line and compilation of qPCR and NGS expression data for selected validated genes; Supplementary File S11: miRNAs detected in specific cells and secreted EVs; Supplementary File S12: Results of differential expression analysis of detected miRNAs among the analyzed cells; Supplementary File S13: Results of differential expression analysis of detected miRNAs among extracellular vesicles secreted by the analyzed cells; Supplementary File S14: Results of differential expression analysis of detected miRNAs among cells and extracellular vesicles; Supplementary File S15: The most significant enriched biological processes and KEGG pathways for target genes associated with downregulated and upregulated miRNAs among cells and extracellular vesicles; Supplementary File S16: Prediction of target genes for differentially expressed miRNA; Supplementary File S17: Results for miRNA target gene overrepresentation in biological processes; Supplementary File S18: Results of miRNA target gene overrepresentation in KEGG pathways.
