# Supplementary material for: Comparative Transcriptome and MicroRNA Profiles of Equine Mesenchymal Stem Cells, Fibroblasts, and Their Extracellular Vesicles
Source: Genes (Basel). 2025 Aug 5;16(8):936. doi: 10.3390/genes16080936 (PMC12386118; doi:10.3390/genes16080936)
Supplement: Supplementary file 1 [file genes-16-00936-s001.zip › Supplementary File S15.pdf]

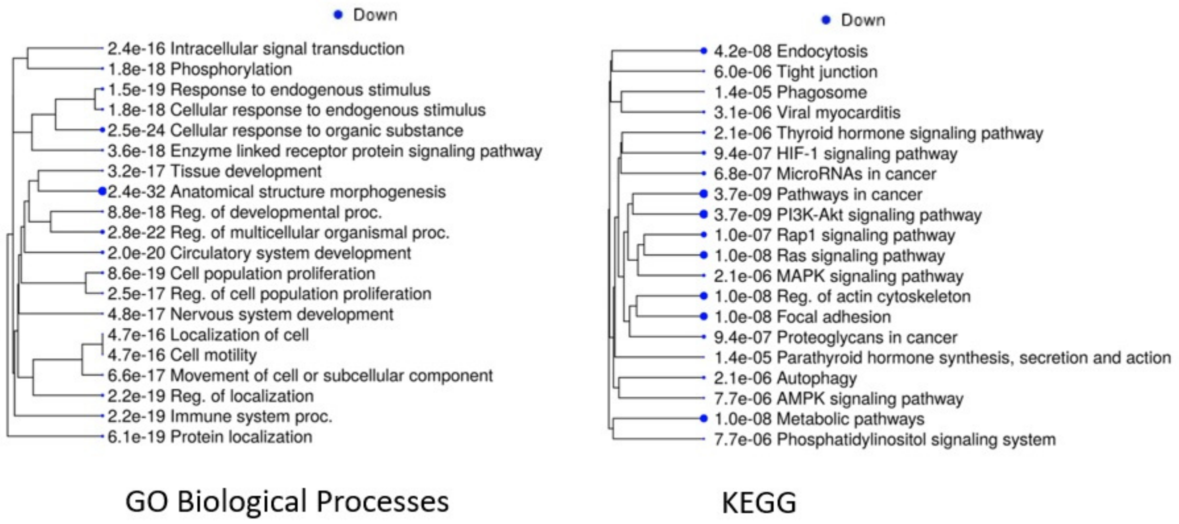

Figure S1. The 20 most significant biological processes and KEGG pathways for target genes associated with downregulated miRNAs in fibroblasts compared to BM-MSC.

The tree represents adjP values, and the size of the dots corresponds to these values.

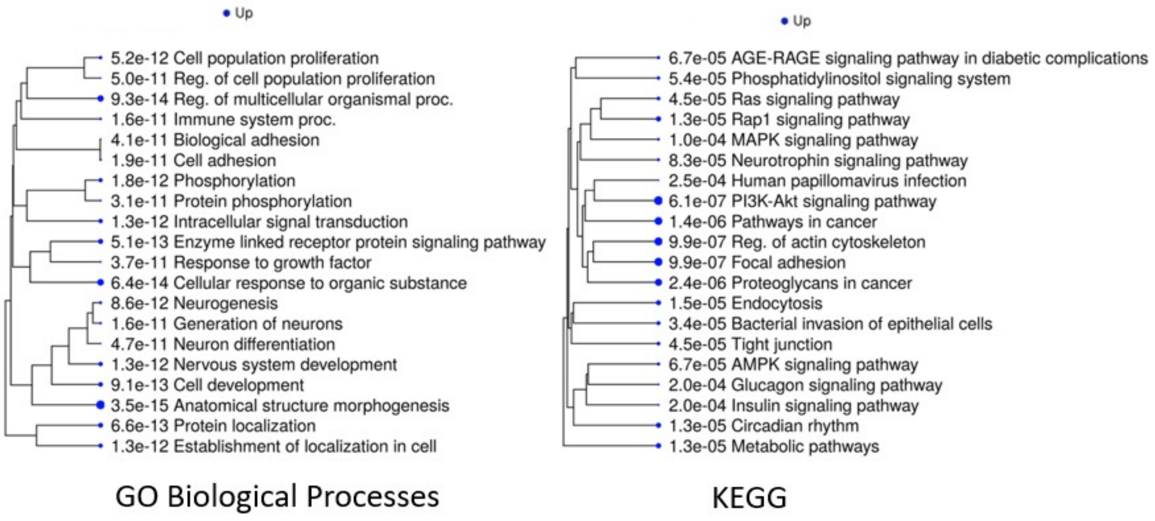

Figure S2. The 20 most significant biological processes and KEGG pathways for target genes associated with upregulated miRNAs in fibroblasts compared to BM-MSC.

The tree represents adjP values, and the size of the dots corresponds to these values.

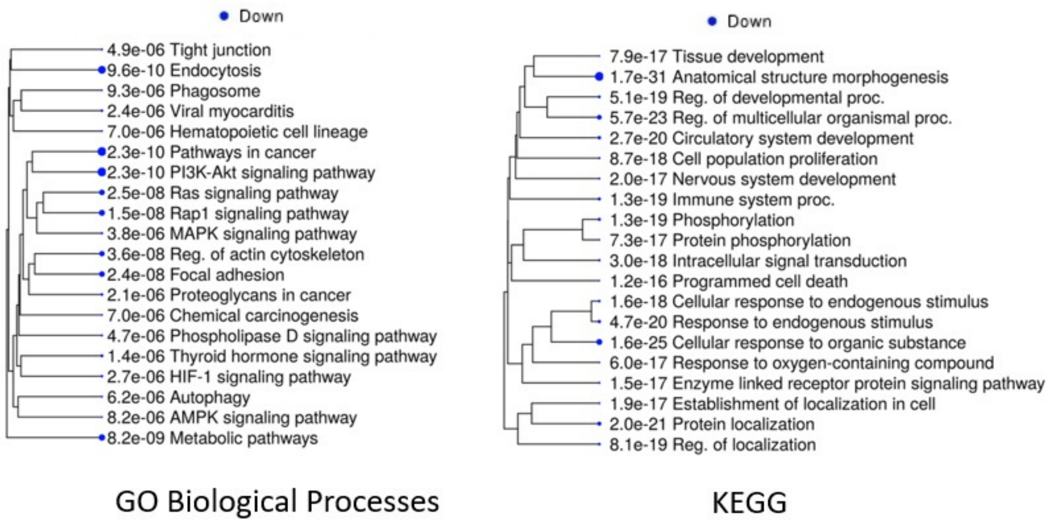

Figure S3. The 20 most significant biological processes and KEGG pathways for target genes associated with downregulated miRNAs in fibroblasts compared to AT-MSC.

The tree represents adjP values, and the size of the dots corresponds to these values.

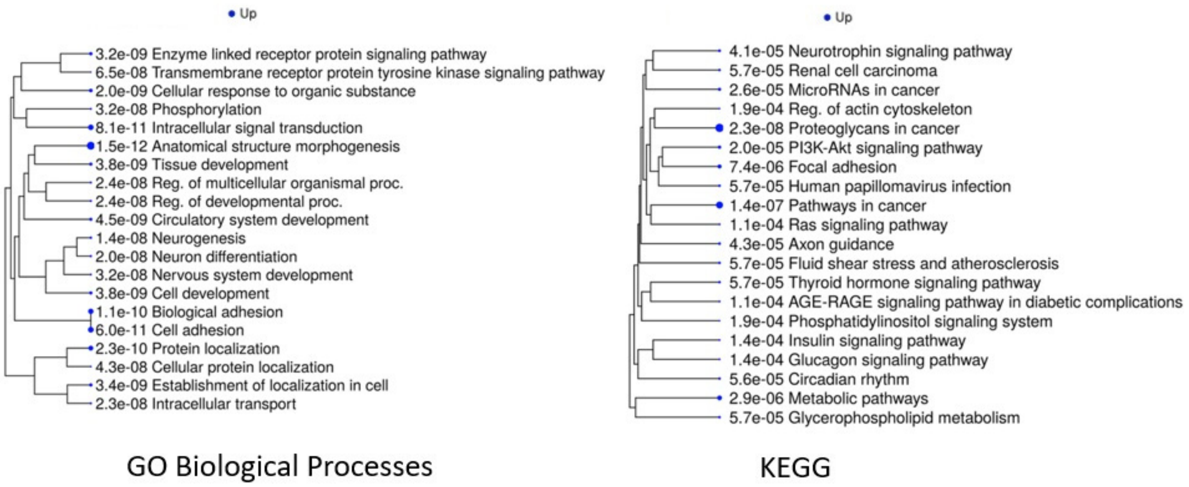

Figure S4. The 20 most significant biological processes and KEGG pathways for target genes associated with upregulated miRNAs in fibroblasts compared to AT-MSC.

The tree represents adjP values, and the size of the dots corresponds to these values.

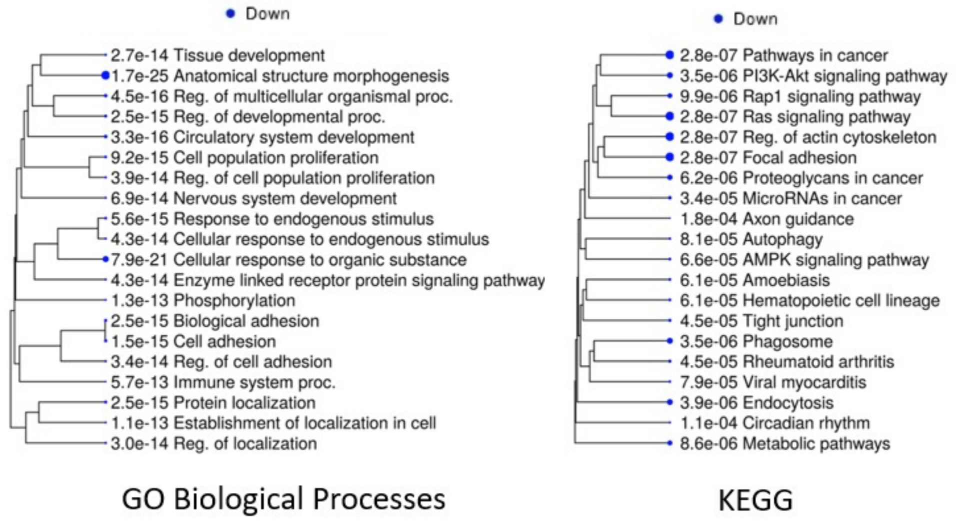

Figure S5. The 20 most significant biological processes and KEGG pathways for target genes associated with downregulated miRNAs in EVs derived from fibroblasts compared to EVs derived from BM-MSC.

The tree represents adjP values, and the size of the dots corresponds to these values.

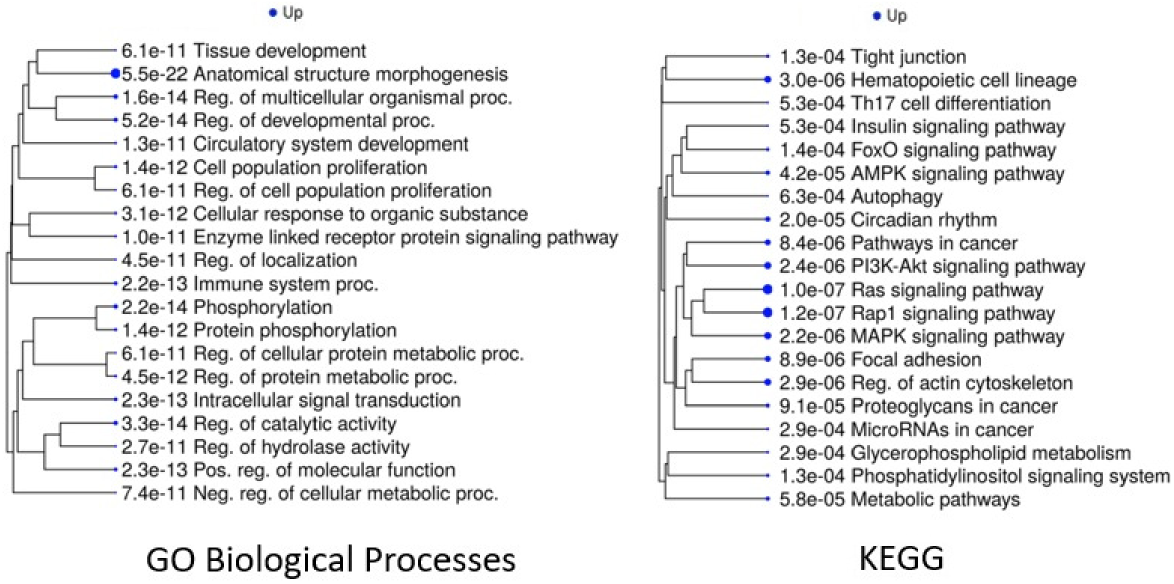

Figure S6. The 20 most significant biological processes and KEGG pathways for target genes associated with upregulated miRNAs in EVs derived from fibroblasts compared to EVs derived from BM-MSC.

The tree represents adjP values, and the size of the dots corresponds to these values.

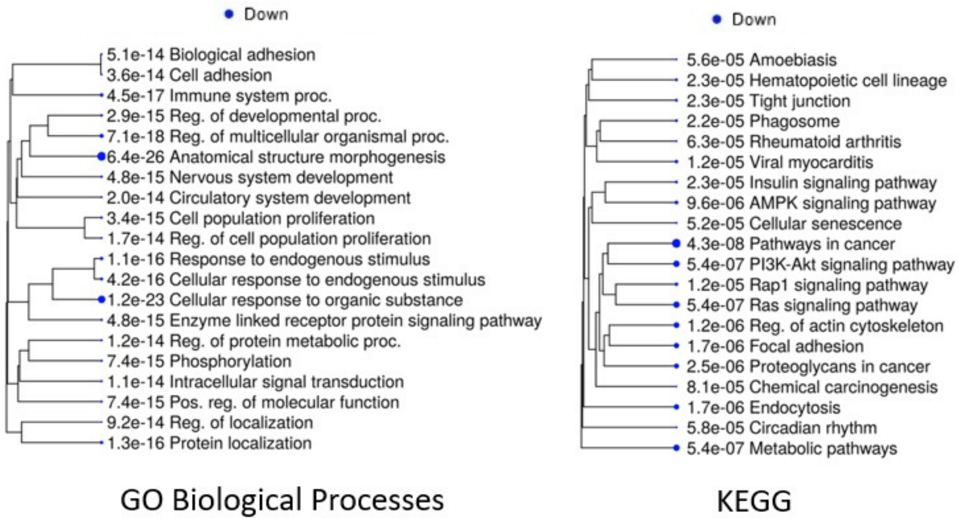

Figure S7. The 20 most significant biological processes and KEGG pathways for target genes associated with downregulated miRNAs in EVs derived from fibroblasts compared to EVs derived from AT-MSC.

The tree represents adjP values, and the size of the dots corresponds to these values.

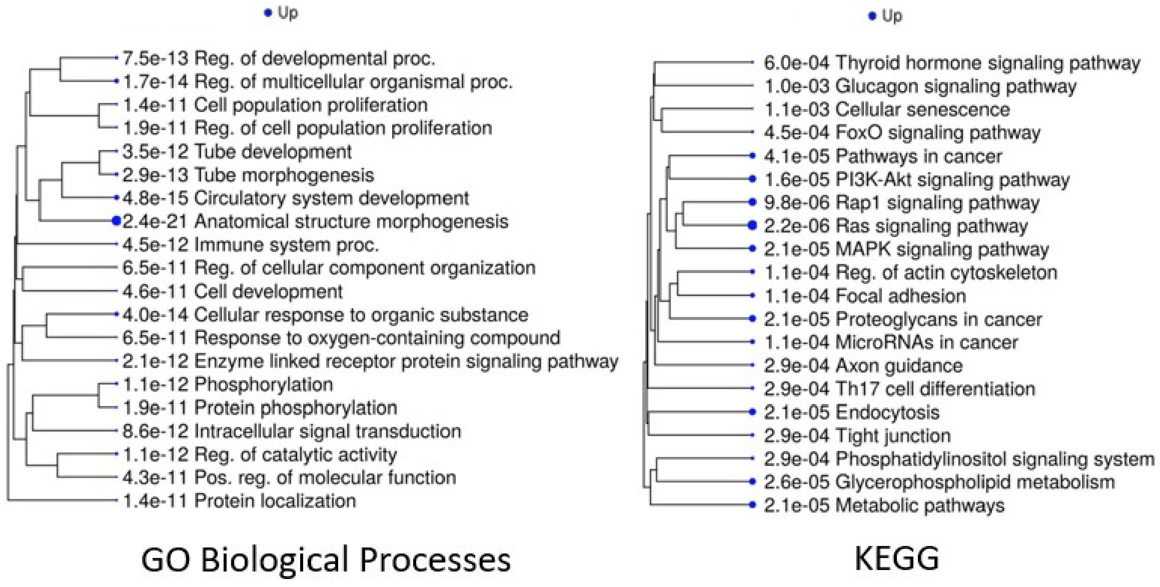

Figure S8. The 20 most significant biological processes and KEGG pathways for target genes associated with upregulated miRNAs in EVs derived from fibroblasts compared to EVs derived from AT-MSC.

The tree represents adjP values, and the size of the dots corresponds to these values.
