# Supplementary material for: Comparative Transcriptome and MicroRNA Profiles of Equine Mesenchymal Stem Cells, Fibroblasts, and Their Extracellular Vesicles
Source: Genes (Basel). 2025 Aug 5;16(8):936. doi: 10.3390/genes16080936 (PMC12386118; doi:10.3390/genes16080936)
Supplement: Supplementary file 1 [file genes-16-00936-s001.zip › Supplementary File S2.pdf]

Analysis of mesenchymal stem cell surface markers by flow cytometry (K1, K2, K3 – adipose-derived MSC; B1, B2, B3 – bone marrow-derived MSC). The figures show control unstained cells, control cells stained with FITC and APC isotypes, and surface antigen MHC II (FL1 PEAK - FITC) and CD90 (FL6 PEAK - APC)

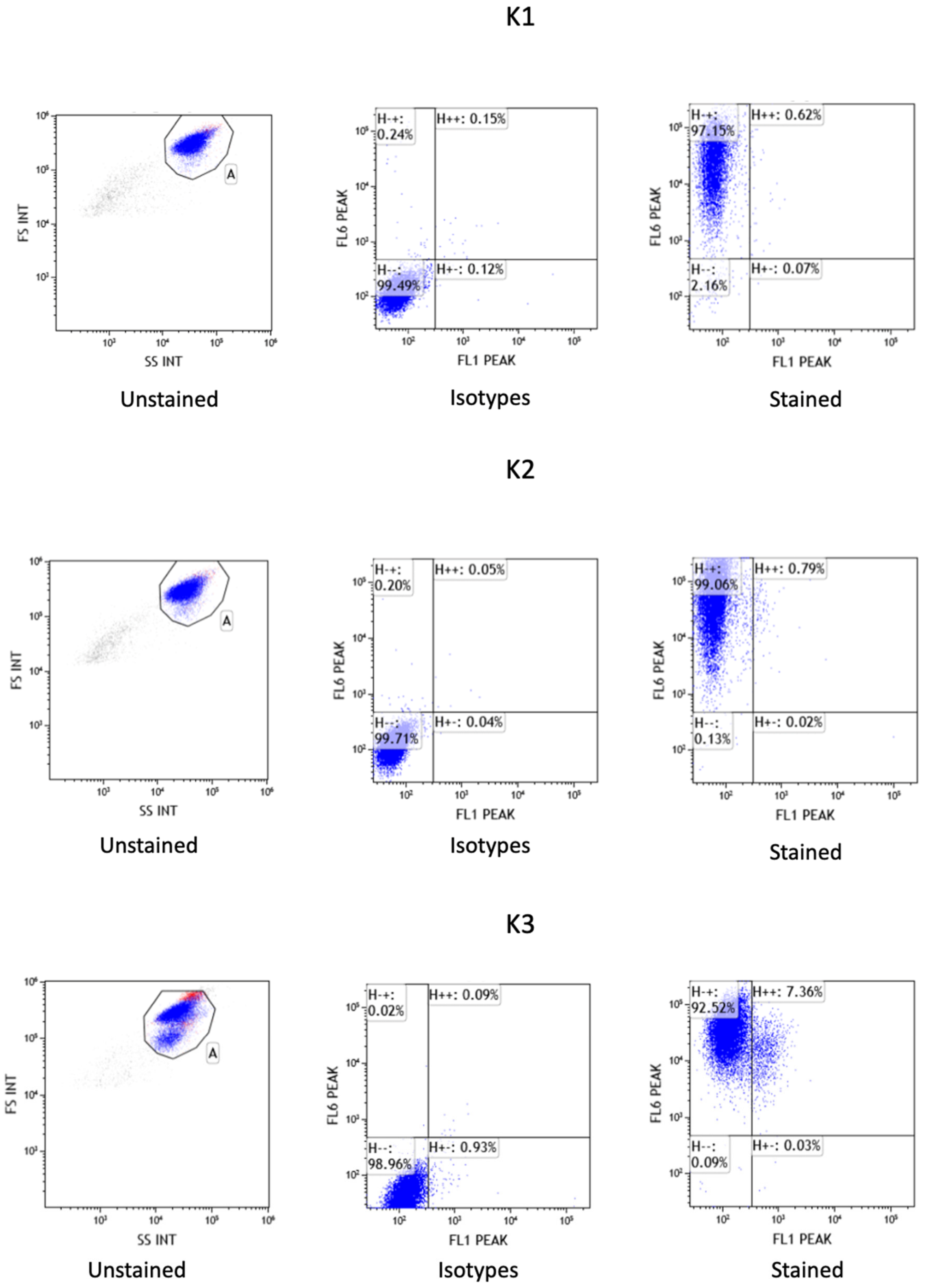

B1

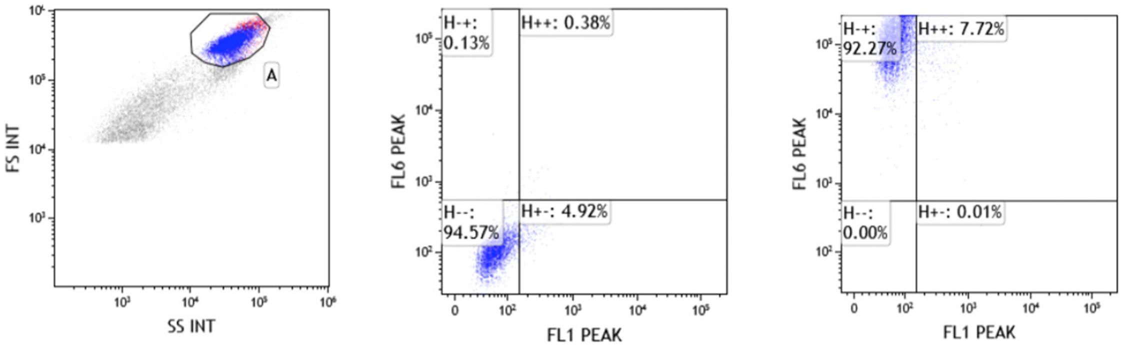

Unstained Isotypes Stained

B2

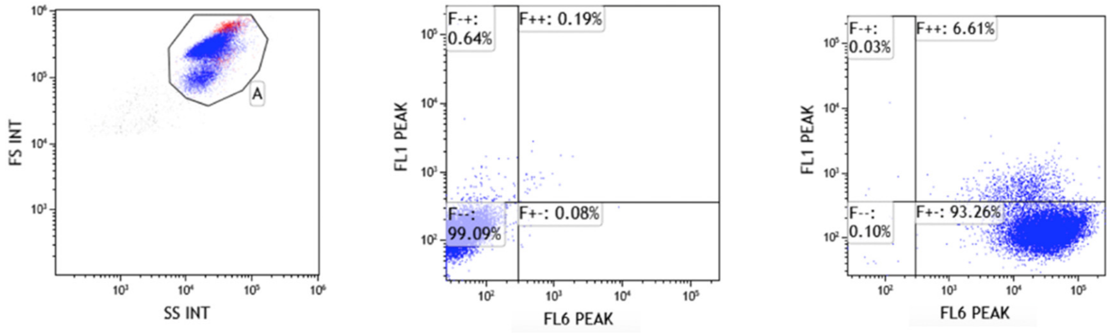

Unstained Isotypes Stained

B3

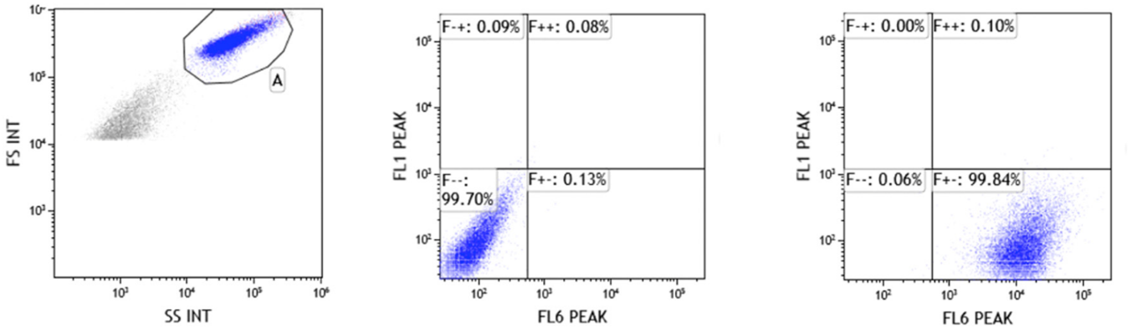

Unstained Isotypes Stained
