# Supplementary material for: Comparative Transcriptome and MicroRNA Profiles of Equine Mesenchymal Stem Cells, Fibroblasts, and Their Extracellular Vesicles
Source: Genes (Basel). 2025 Aug 5;16(8):936. doi: 10.3390/genes16080936 (PMC12386118; doi:10.3390/genes16080936)
Supplement: Supplementary file 1 [file genes-16-00936-s001.zip › Supplementary File S3.pdf]

Nanoparticle tracking analysis (NTA). Representative analysis of an EVs by NanoSight NS500 showing the concentration of EV-MSCs and EV-fibroblasts in particles per mL and the diameter of the EV-MSCs and EV-fibroblasts in nanometers (nm). *The sample was analyzed in triplicate and red lines are standard error bars.*

F1

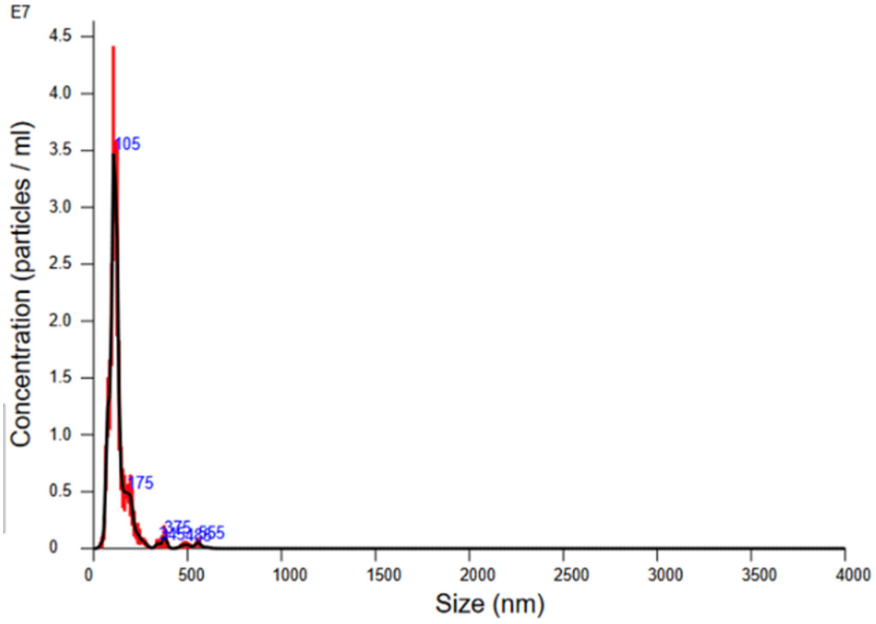

F2

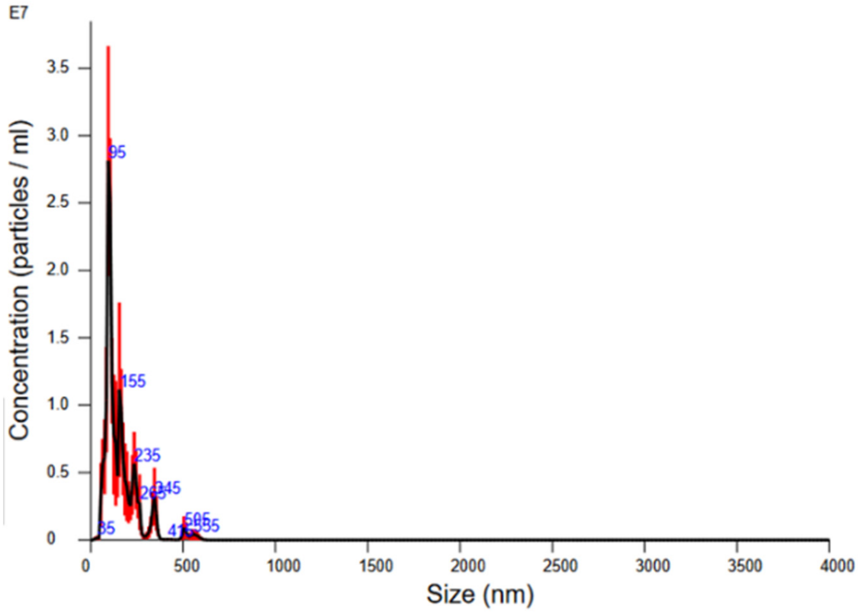

F3

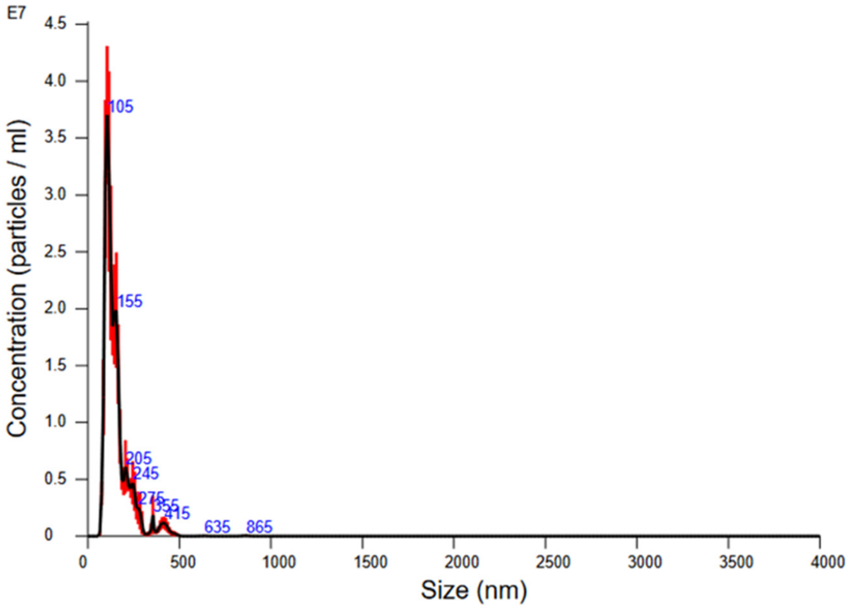
E7
Concentration (particles / ml)
Size (nm)
105
155
205
245
275
355
415
635
865
0
0.5
1.0
1.5
2.0
2.5
3.0
3.5
4.0
4.5
500
1000
1500
2000
2500
3000
3500
4000

K1

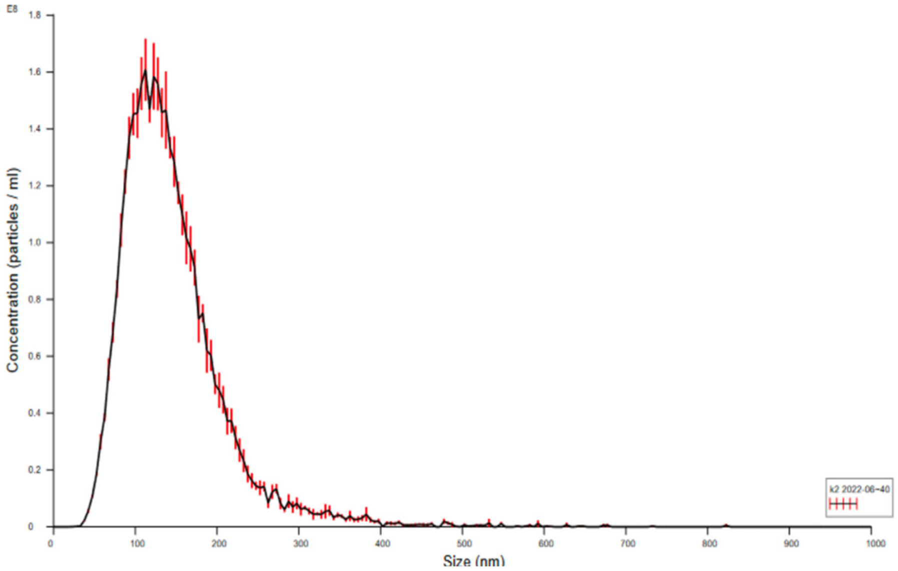
E8
Concentration (particles / ml)
Size (nm)
k2 2022-06-40
0
0.2
0.4
0.6
0.8
1.0
1.2
1.4
1.6
1.8
100
200
300
400
500
600
700
800
900
1000

K2

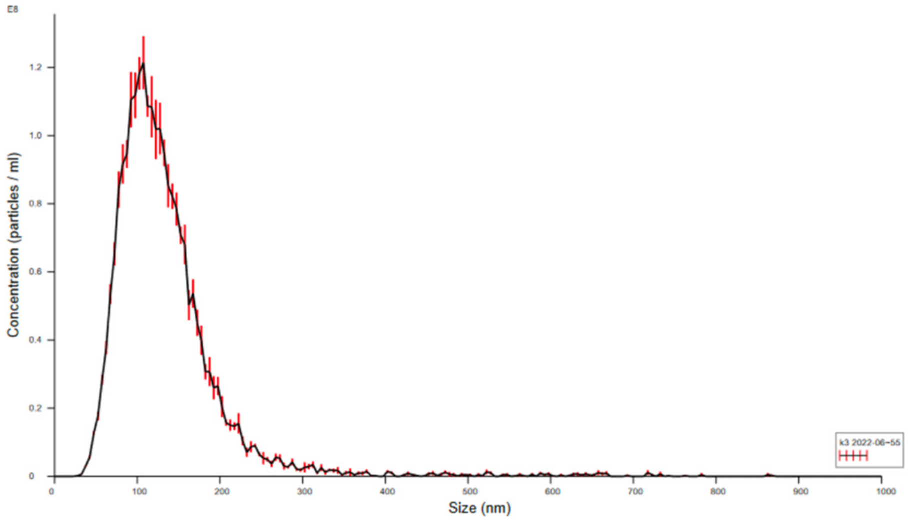
E8
1.2
1.0
0.8
0.6
0.4
0.2
0
Concentration (particles / ml)
0
100
200
300
400
500
600
700
800
900
1000
Size (nm)
k3 2022-06-55

K3

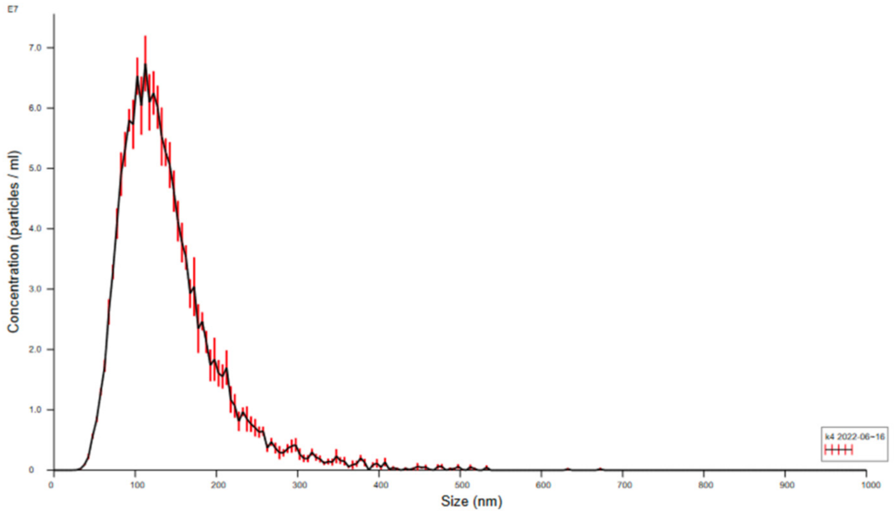
E7
7.0
6.0
5.0
4.0
3.0
2.0
1.0
0
Concentration (particles / ml)
0
100
200
300
400
500
600
700
800
900
1000
Size (nm)
k4 2022-06-16

B1

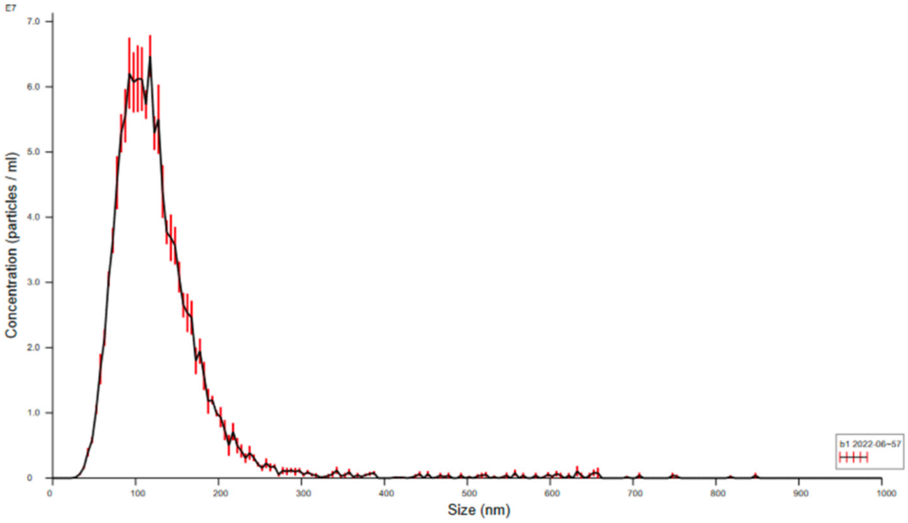
E7
Concentration (particles / ml)
7.0
6.0
5.0
4.0
3.0
2.0
1.0
0
0
100
200
300
400
500
600
700
800
900
1000
Size (nm)
b1 2022-06~57

B2

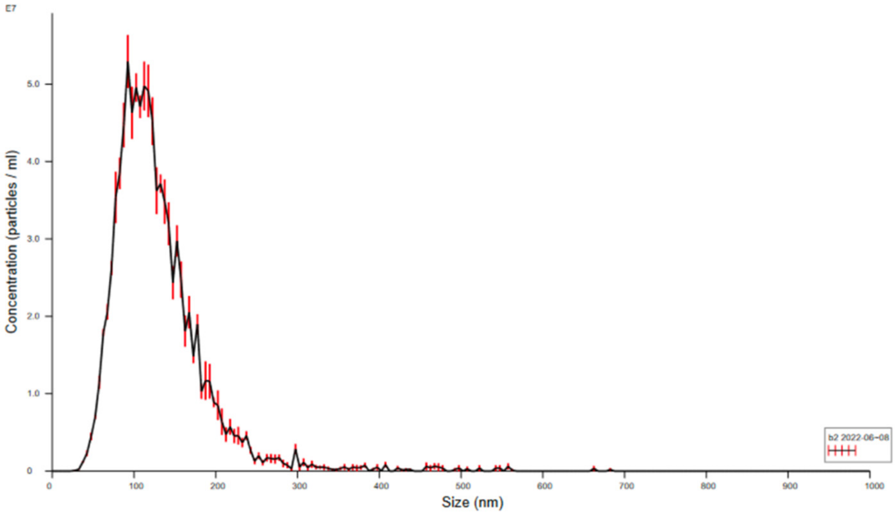
E7
Concentration (particles / ml)
5.0
4.0
3.0
2.0
1.0
0
0
100
200
300
400
500
600
700
800
900
1000
Size (nm)
b2 2022-06~08

B3

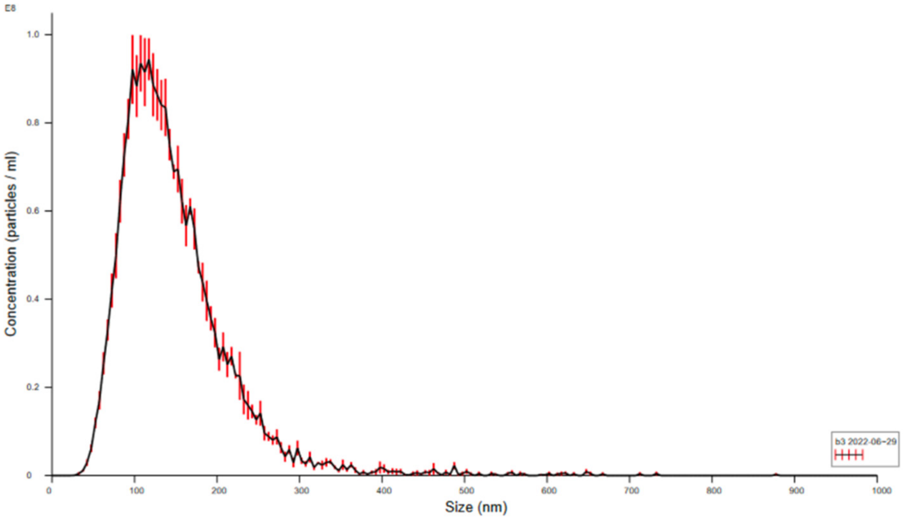
E8
1.0
0.8
0.6
0.4
0.2
0
Concentration (particles / ml)
0
100
200
300
400
500
600
700
800
900
1000
Size (nm)
b3 2022-06-29
