# Supplementary material for: Comparative Transcriptome and MicroRNA Profiles of Equine Mesenchymal Stem Cells, Fibroblasts, and Their Extracellular Vesicles
Source: Genes (Basel). 2025 Aug 5;16(8):936. doi: 10.3390/genes16080936 (PMC12386118; doi:10.3390/genes16080936)
Supplement: Supplementary file 1 [file genes-16-00936-s001.zip › Supplementary File S4.pdf]

High-resolution flow cytometry of EV samples. Representative dot plots of EV-MSCs (K1, K2, K3 – adipose tissue; B1, B2, B3 – bone marrow) and EV-fibroblasts (F1, F2, F3-fibroblasts), collected with the A60-Micro-PLUS cytometer. The percentage of positive objects for the analyzed markers is shown in red gates. LALS – large-angle light scatter parameter, corresponding to the relative size of the analyzed particles.

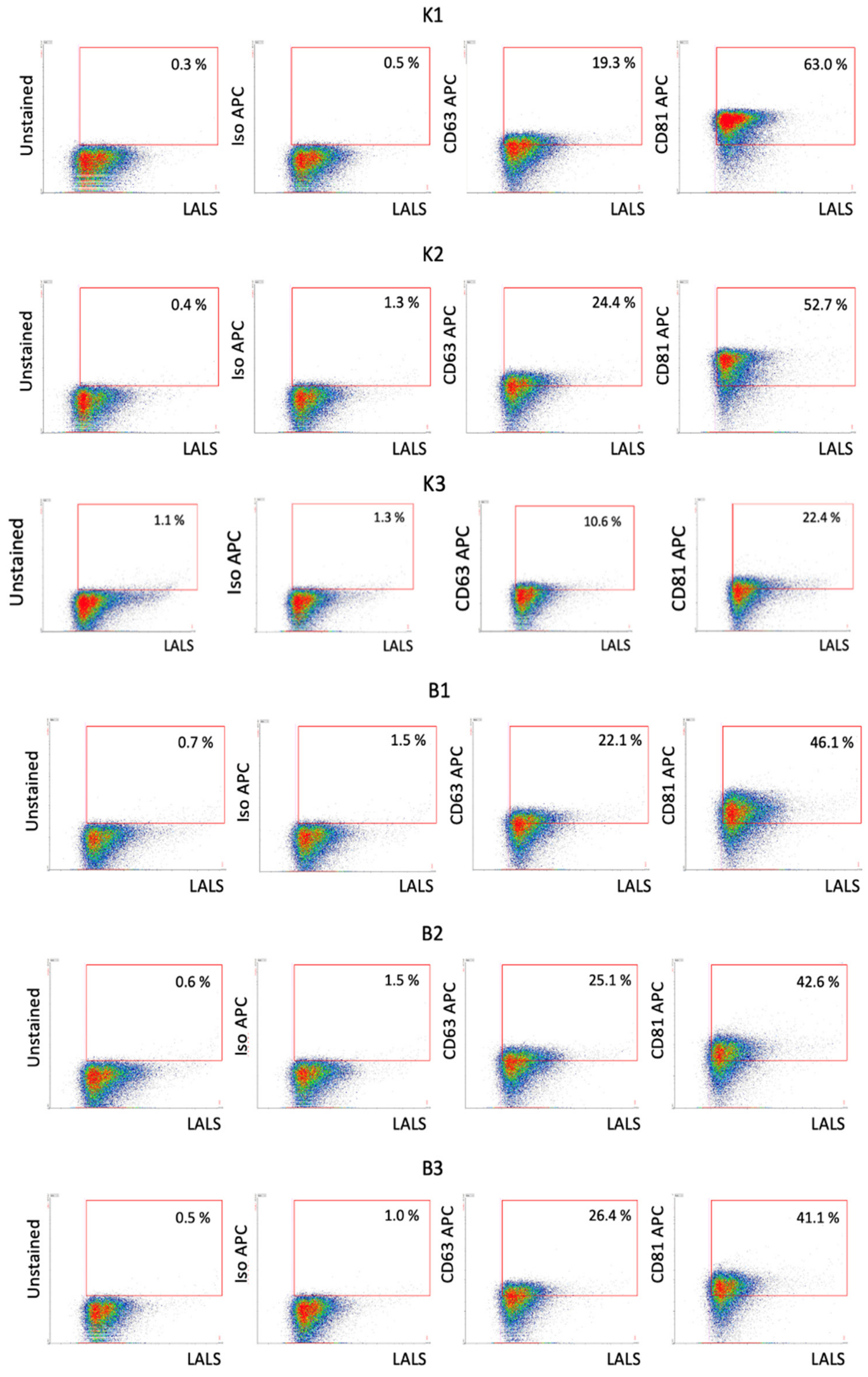

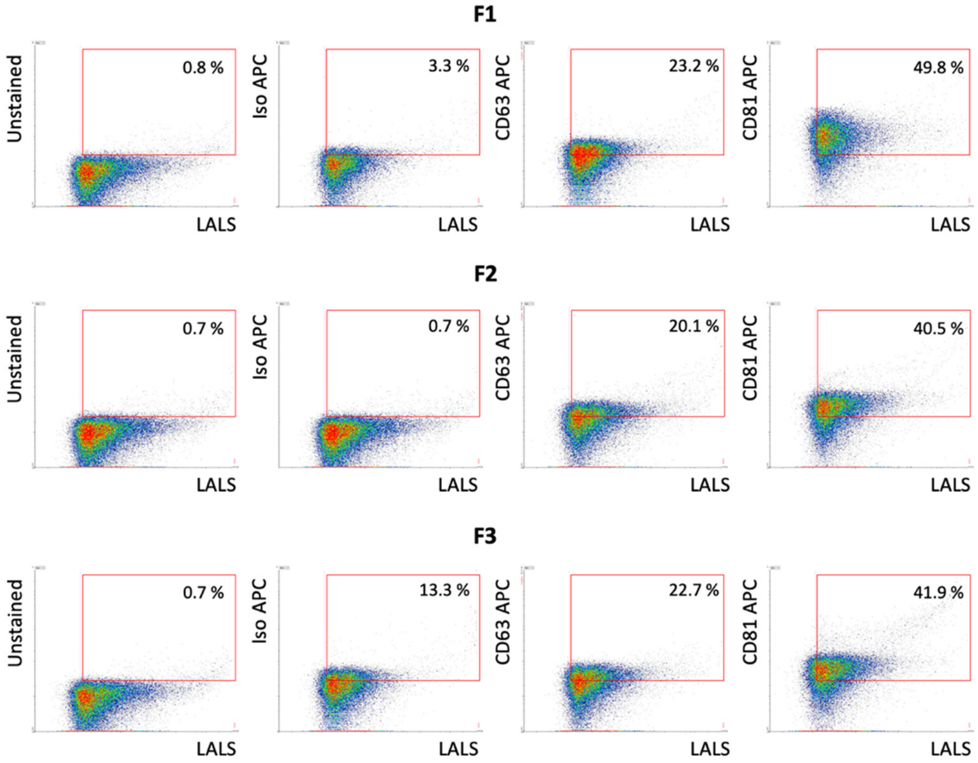

F1
Unstained
0.8 %
LALS
Iso APC
3.3 %
LALS
CD63 APC
23.2 %
LALS
CD81 APC
49.8 %
LALS
F2
Unstained
0.7 %
LALS
Iso APC
0.7 %
LALS
CD63 APC
20.1 %
LALS
CD81 APC
40.5 %
LALS
F3
Unstained
0.7 %
LALS
Iso APC
13.3 %
LALS
CD63 APC
22.7 %
LALS
CD81 APC
41.9 %
LALS
